# Green AI architectures: Navigating the security–sustainability paradox in critical infrastructure protection

**DOI:** 10.1016/j.ese.2026.100697

**Published:** 2026-04-06

**Authors:** JungMin Lee, Amir Saman Tayerani Charmchi, Fatemeh Ghobadi, Myeong In Kim

**Affiliations:** a Land and Housing Research Institute, Smart Climate Environment Research Center, Daejeon, Republic of Korea; b Digital Twin and Artificial Intelligence Research Lab, Digital Integration Department, Onpoom Corp. R&D Center, Seoul, 07222, Republic of Korea

**Keywords:** Cost-effectiveness analysis, Cyber–physical security, Green AI, Reconstruction-based anomaly detection, Sustainable computing

## Abstract

Critical cyber–physical infrastructure, such as urban water distribution systems, underpins public health, economic stability, and environmental sustainability, yet faces escalating threats from sophisticated cyber–physical attacks that evade traditional defenses. Deep-learning-based reconstruction models offer the adaptability needed to detect unseen anomalies but impose prohibitive computational and environmental costs, creating an unresolved tension between security and sustainability. While PCA–deep-learning hybrids are widely used, their architectural configurations for anomaly detection have remained naive and unquantified in terms of real-world resource demands. This study demonstrates that among five novel PCA–autoencoder configurations evaluated across two challenging water distribution datasets, architectural synthesis dictates both detection robustness and sustainability, with operational efficiency varying by over an order of magnitude. An integrated model (PCA-D) achieves strong anomaly detection at a cost-effectiveness ratio of 2.62 J per true positive—nearly four times better than the most robust hybrid—while naive wrapper hybrids miss over 77% of threats. The proposed framework converts measured computational loads into annual energy, carbon, and water footprints, revealing that the most detection-robust model is not the most sustainable. These results establish a unifying cost-effectiveness metric and a key design principle: integrated statistical–deep-learning architectures enable genuinely green AI that secures critical infrastructure without incurring excessive environmental burden.

## Highlights

• Model architecture, not size alone, dictates AI's environmental costs in cyber-physical security.
• Novel CER quantifies cost per detected threat, unifying security and sustainability.
• Integrated PCA–DL delivers strong detection at a fraction of wrapper and standalone DL costs.
• Top hybrid achieves high robustness at four times the cost of the most efficient model.
• Framework blueprints sustainable AI for critical infrastructure without planetary compromise.

## Graphical abstract

**Figure:**
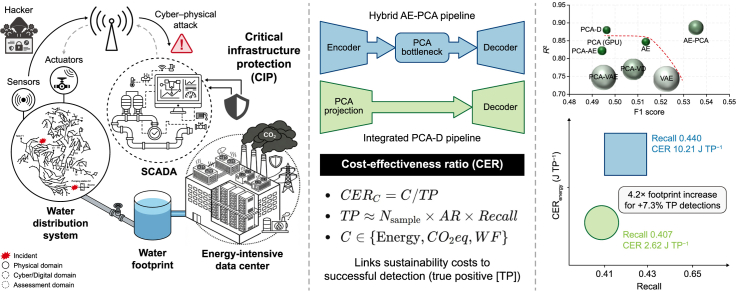

## 1 Introduction

Critical infrastructure protection (CIP) is fundamental to the sustainability and resilience of modern urban centers [1]. As a foundational example, the integrity of water distribution systems (WDS) is a direct prerequisite for public health, economic stability, and environmental sustainability [2]. However, this critical infrastructure is confronting a dual crisis. A legacy of physical decay results in an estimated annual global loss of 126 billion cubic meters of treated water [3]. In the United States alone, this problem is particularly acute, with a water main break reported approximately every 2 min [4]. In response, utilities organizations have increasingly pursued digital transformation [5], integrating supervisory control and data acquisition (SCADA) systems and edge devices to evolve WDS into sophisticated cyber–physical systems (CPS) [6]. While this digitalization is essential for sustainability, it introduces a profound vulnerability: the expanded digital attack surface exposes the physical control of WDS to malicious cyber–physical threats [7], creating a fragile nexus in which addressing one crisis may inadvertently create another.

Such cyber–physical threats are no longer theoretical. Real-world incidents, including the Maroochy Shire sewage SCADA compromise (Queensland, Australia), the Oldsmar water treatment plant intrusion (Florida, the United States), and recent attacks targeting municipal water utilities in Texas (the United States), demonstrate the emergence of sophisticated threats that can evade traditional security mechanisms [8,9]. Rather than simple intrusions, these attacks manipulate operational parameters in ways designed to mimic normal system behaviors [10] such as subtly adjusting chemical dosages within permissible ranges or incrementally altering pump states over extended periods [11]. Such stealthy manipulations introduce anomalies that can only be identified contextually through their deviation from a complex, nonlinear baseline of normal operational conditions [12], making them precisely the type of previously unseen threats that static, rule-based security systems inherently fail to detect [13,14]. Consequently, CIP necessitates shifting from basic anomaly detection toward artificial intelligence (AI)-driven methods [15], particularly reconstruction-based approaches that enforce normal system behavior [16]. Specifically, deep learning (DL) models, which are often employed in an unsupervised learning paradigm, have emerged as the state-of-the-art solution for achieving this adaptability [17,18]. However, their implementation introduces a second, equally profound paradox: the solution to a critical sustainability threat comes with its own prohibitive and escalating environmental costs [19], creating a direct tension between security and sustainability.

Recent literature indicates that in response to sophisticated nonlinear cyber–physical threats, autoencoder (AE) networks have emerged as promising DL-based reconstruction methods for unseen anomaly detection [20]. Operating under an unsupervised principle, these neural networks learn to encode high-dimensional sensor data into a compressed latent representation of normal system behavior [21], identifying previously unseen anomalies as samples whose decoded outputs deviate substantially from the inputs (i.e., high reconstruction error) [22]. Recently, the field has expanded beyond basic AE structures and variational variants (VAEs) to more sophisticated spatial, temporal, and spatiotemporal architectures, including graph neural networks (GNNs) [23] and attention-based networks [24,25]. These sophisticated architectures are increasingly viewed as leading approaches for unseen anomaly detection, a shift documented in recent systematic reviews [26]. However, a critical examination of the literature reveals that the immense cost associated with such effectiveness is often overlooked [[27], [28], [29]]. When deployed at the massive scale required for urban infrastructure, this computational cost translates into a full-spectrum environmental burden of immense energy, water, and carbon footprints [30,31].

This burden is particularly critical given the explosive growth in AI-driven computational workloads [32]. Globally, data center energy demand is projected to more than double by 2030, with approximately 70% of this growth driven directly by Accelerated servers [33]. By 2030, these AI-optimized servers alone are anticipated to consume ∼432 TWh annually [34], exceeding the annual solar electricity currently generated by the European Union (∼369 TWh in 2025) [35]. This energy demand exacerbates grid stability risks and contributes directly to increased carbon footprints [36], as grid stabilization efforts may increase reliance on fossil fuels [37]. For example, training an attention-based model as large as GPT-3 can produce as much carbon as 123 gasoline-powered vehicles emit in a year [38]. Water usage represents another critical yet frequently overlooked dimension. Hyperscale data centers routinely withdraw 0.001 to 3 L per kWh of workload for cooling [39], and can indirectly consume 0.008 to 5.17 L of fresh water per kWh of electricity when upstream power generation is considered [40]. This paradoxical sustainability crisis must be managed by any society pursuing smart, sustainable cities. Therefore, the central challenge in deploying AE/VAE models to protect critical infrastructure is not simply improving detection effectiveness, but rather navigating this paradox to reduce both the tangible and intangible costs of models.

Addressing this cost-effectiveness tradeoff necessitates adopting the emerging green AI paradigm, which elevates resource efficiency from a secondary consideration to a primary design objective, on par with predictive accuracy [41]. Historically, research in this area has prioritized model-centric optimizations ranging from network compression techniques such as pruning and quantization [42] to the development of efficient neural architecture search methods [43]. Complementing these model-centric efforts are data-centric strategies [44] such as efficient techniques to simplify input complexity [45]. While these post-hoc optimizations are valuable, a more foundational strategy achieves inherent efficiency at the design stage by architecturally synthesizing data-centric statistical methods with expressive AE models. In this context, synthesizing the computational efficiency and robust statistical properties of data-driven methods such as principal component analysis (PCA) with the expressive capabilities of DL represents an under-investigated yet potentially transformative approach [46,47]. Previous studies have frequently synthesized AE with PCA across various fields, including image generation [48], remote sensing [49], artifact suppression [50], medical diagnostics [51], operation control [52], and fraud analysis [53,54]. These studies have demonstrated that this architectural synthesis can practically mitigate computational complexity while maintaining or even enhancing model performance.

However, a critical examination of the CPS security literature reveals that PCA is predominantly applied in a simplistic manner, serving primarily as a preprocessing step aimed at basic dimensionality reduction. This shallow approach is prevalent across domains such as intrusion detection [[55], [56], [57], [58]] and fault prognosis [59,60], and contrasts sharply with the more sophisticated architectural synthesis explored in image generation and artifact suppression [50]. For example, Pham et al. effectively used PCA-derived principles to systematically organize and disentangle complex latent spaces, demonstrating a richer, more analytically rigorous application of PCA-AE in generative networks [48]. Although the shallow PCA-based approach has yielded nominally acceptable results on selected benchmarks in conventional applications [55,60], it remains fundamentally inadequate for AI-driven CPS security in smart cities. Most critically, this study argues that within CIP, the core assumption of PCA that high-variance components carry the most relevant information is largely inverted in unseen anomaly detection scenarios, where subtle, low-variance deviations often signify the most severe and impactful threats.

Despite extensive research into DL-based anomaly detection and growing awareness of AI's environmental burden, a critical gap persists at the nexus of CIP, AI-driven CPS, and sustainable cities. The literature is markedly bifurcated, with one body of work prioritizing detection effectiveness with little regard for prohibitive computational costs, while another examines AI's macro-level sustainability costs, devoid of the CIP context. Consequently, there is a conspicuous absence of empirical research connecting the architectural design of anomaly detection models with their real-world computational and environmental costs. This divide is particularly acute for hybrid PCA–DL approaches, where their application to anomaly detection has been architecturally naive and limited to superficial preprocessing. This approach overlooks the fundamental inversion of PCA's core assumption in CPS security, particularly in critical infrastructure such as WDS, where the subtle, low-variance signatures of novel anomalies can represent the most catastrophic threats. Therefore, a systematic and holistic investigation of various PCA–DL configurations, evaluated through a rigorous cost-effectiveness framework, is not merely a gap in the literature but a critical and unmet need for operationalizing secure, scalable, and sustainable AI in smart city infrastructures.

This study aims to address this critical research gap by proposing and comprehensively evaluating novel PCA–DL configurations through two design strategies: hybridization and integration. This research provides three key empirical advancements. First, it provides a comparative analysis of five novel PCA–DL architectures against established benchmarks on two distinct WDS datasets that represent different attack scenarios. Second, it develops a multi-stage cost analysis framework that translates measured computational loads into sustainability costs, including annual energy requirements, carbon emissions, and water consumption. Finally, it formulates and applies a novel cost-effectiveness ratio (CER) as a unifying metric that quantifies the resource cost per correctly identified anomaly. By integrating these elements, this research answers two central questions. First, how do these novel architectures impact detection effectiveness against sophisticated cyber–physical attacks in a real-world WDS? Second, and more critically, what are their real-world costs, and how can a holistic framework be used to select the most genuinely cost-effective solution for securing the sustainable cities of the future?

The remainder of this paper details the proposed architectures and methods (Section 2), describes a comprehensive evaluation framework (Section 3), presents empirical results and discussion (Section 4), and offers concluding remarks (Section 5).

## 2 Methods and techniques

This section details the methodological and architectural foundations of this study, beginning with the principles of reconstruction-based anomaly detection. It then provides a concise technical overview of the two core components under investigation: the linear statistical method of PCA and the nonlinear AE and VAE networks. Finally, it presents the core architectural contribution of this study: the design and rationale for five distinct PCA–DL configurations, organized under two competing design strategies: hybridization and integration.

### 2.1 Reconstruction-based anomaly detection

**Fig. 1:**
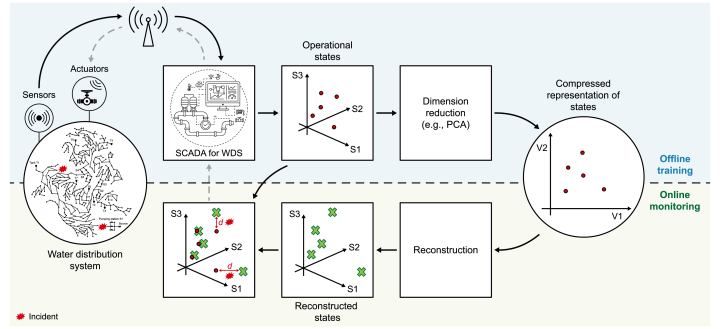
Schematic framework of the reconstruction-based anomaly detection applied to water distribution systems (WDS). PCA, principal component analysis; SCADA, supervisory control and data acquisition.

The core methodological approach of this study is reconstruction-based anomaly detection, a paradigm well-suited to the complex, high-dimensional data streams generated by the SCADA systems that govern modern WDS [61]. The general framework (Fig. 1) operates on the principle that a model trained exclusively on normal operational data from a CPS will learn to accurately reproduce that data. The framework functions in two phases. First, in an offline training phase, a dimensionality reduction model learns a compressed, latent representation of the system's normal behavior. The model is optimized to minimize a reconstruction loss function, effectively creating a high-fidelity mapping of the WDS's normal operational manifold. Second, in the online operational phase, the trained model is used to reconstruct new, unseen SCADA data. Anomalies, such as those caused by cyber–physical attacks, deviate from learned normal patterns and cannot be reconstructed accurately, resulting in a high reconstruction error. This error is formally quantified using a metric such as the squared prediction error (SPE, s), which is calculated as

(1) $$s=e^{⊤}e={\left(X-\overset{´}{X}\right)}^{⊤}\left(X-\overset{´}{X}\right)$$

where e represents the residual vector between the input data vector X and reconstructed output $\overset{´}{X}$. A predefined statistical threshold ($T_{\text{SPE}}$) is then set as the q th quantile of s computed on the training data. Any observation with a reconstruction error above this threshold is identified as an anomaly.

### 2.2 Dimensionality reduction techniques

This study investigates two distinct classes of dimensionality reduction techniques for managing high-dimensional data streams from WDSs.

#### 2.2.1 Statistical algorithm: PCA

Principal component analysis (PCA) is a linear dimensionality reduction technique that transforms a set of correlated variables into a set of principal components (PCs) as new uncorrelated variables [62,63]. This transformation is defined such that the first PC accounts for the largest possible variance in the data, and each succeeding component has the highest variance possible under the constraint that it is orthogonal to the preceding PCs. In this study, the transformation is performed by decomposing the input data matrix X, using singular value decomposition (SVD) as follows:

(2) $$X=U\Sigma V^{⊤}$$

where U and V are orthogonal matrices of the left and right singular vectors, respectively, and Σ is a diagonal matrix containing the singular values of X. The data are projected onto a lower-dimensional space of k PCs through a linear transformation, yielding the matrix of scores Z as:

(3) $$Z_{k}=XV_{k}$$

where $V_{k}$ contains the first k right-singular vectors (the principal directions). The original data are then approximated by reconstruction via the following inverse transformation:

(4) $$\overset{´}{X}=Z_{k}V_{k}^{⊤}$$

This combination of low computational overhead and the ability to impose a linear structure on high-dimensional data makes PCA a foundational technique for developing more efficient DL architectures within CPS in a WDS.

#### 2.2.2 DL algorithms

The primary objective of this study is not simply to incrementally improve the state-of-the-art detection score but to conduct a comparative analysis of the computational cost and sustainability implications of different architectural strategies. For this purpose, using well-understood, foundational DL architectures such as vanilla AEs and VAEs is a more rigorous and interpretable choice. They provide a clear and reliable baseline against which the costs and benefits of different design strategies can be evaluated unambiguously. Although advanced spatiotemporal architectures—ranging from convolutional neural networks and long short-term memory-based approaches for spatial and temporal dependencies [[55], [56], [57], [58]] to GNNs and Transformer variants for topological and long-range dependencies [23,25]—have demonstrated high efficacy in CPS, their inclusion would introduce confounding variables. Such complexity would make it impossible to isolate the specific impact of synthesizing linear and nonlinear components, which is the core methodological goal of this study. Therefore, the focus on foundational architectures is a deliberate choice to ensure the internal validity and interpretability of the findings.

**Fig. 2:**
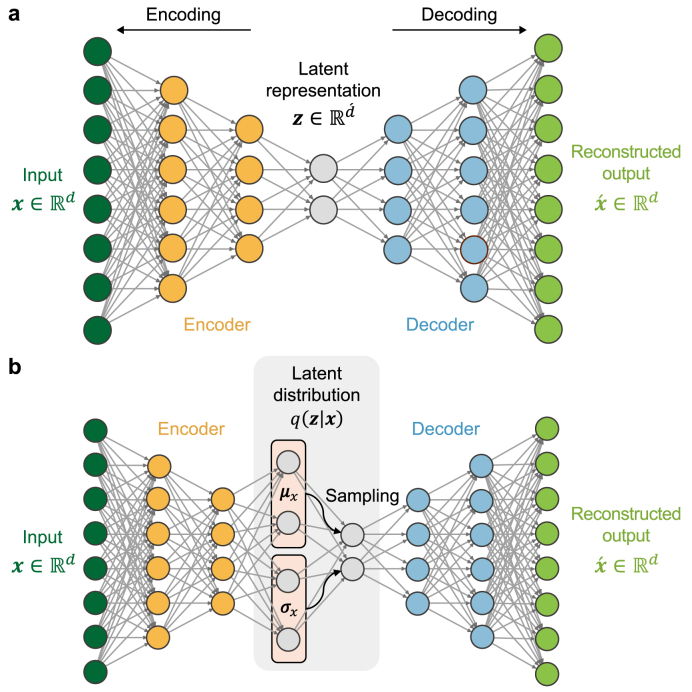
Schematic representation of feedforward neural networks for reconstruction. **a,** Autoencoder (AE); **b**, Variational autoencoder (VAE).

An AE is a feedforward neural network containing two main components (Fig. 2a): an encoder network that maps the high-dimensional input data $x\in R^{d}$ to a lower-dimensional latent representation $z\in R^{\overset{´}{d}}$ and a decoder network that reconstructs the output $\overset{´}{x}\in R^{d}$ from z [64]. This deep network is trained via unsupervised learning by minimizing the reconstruction loss between the input and output. The loss function L(·) is typically defined as follows:

(5) $$L_{\text{AE}}=\frac{1}{n}\sum_{i=1}^{n}{\left\|x_{i}-{\overset{´}{x}}_{i}\right\|}^{2}$$

where $L_{\text{AE}}$ is the loss function for the AE, n is the number of training samples, and $x_{i}$ is the i th training input. This process forces the network to learn a compressed representation of the most salient features of the data, making it a powerful tool for capturing complex, nonlinear relationships.

The VAE is a generative extension of the AE that employs a probabilistic approach to the latent space (Fig. 2b) [64]. Instead of mapping the input to a single point, the VAE encoder outputs separate vectors representing the mean μ and variance σ of a probability distribution. A latent vector z is then sampled from this distribution and passed to the decoder. This stochasticity enables the VAE not only to reconstruct inputs but also to generate novel data. To enforce this structure, the VAE is trained to minimize a function L(·) composed of two terms, namely a reconstruction loss similar to $L_{\text{AE}}$ and a Kullback–Leibler (KL) divergence term ($D_{\text{KL}}$) that regularizes the latent space by forcing the learned distribution to be similar to a standard normal distribution [65].

(6) $$L_{\text{VAE}}=E_{q\left(z|x\right)}\left[\log\;p\left(x|z\right)\right]-D_{\text{KL}}\left(q\left(z|x\right)\|p\left(z\right)\right)$$

Here, the first term is the expected reconstruction loss and the second is the KL divergence between the approximate posterior q(z|x) and prior p(z). This regularization helps prevent overfitting and encourages a more structured and continuous latent space.

### 2.3 Proposed method and PCA–DL configurations

The primary contribution of this study is a rigorous evaluation framework for quantifying the security–sustainability tradeoff in AI-driven CIP. To validate this framework across a representative spectrum of complexity, this section details the design of five distinct graphics processing unit (GPU)-accelerated PCA–DL configurations. These configurations are developed under two competing design strategies: hybridization and integration. The hybridization strategy cascades complete PCA and DL pipelines sequentially, creating multi-stage models that process data cooperatively. In contrast, the integration strategy streamlines the architecture by eliminating redundant components, such as by replacing the DL encoder entirely with a direct PCA projection to create a more compact and computationally efficient model. These strategies offer tunable tradeoffs between model complexity and detection performance, providing a diverse basis for evaluating the proposed CER metric.

**Fig. 3:**
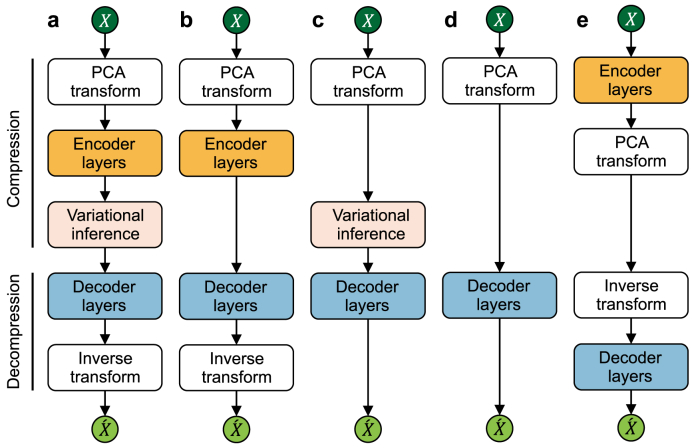
Visual representation of the five proposed configurations based on hybridization and integration of principal component analysis (PCA) with deep learning (DL). **a**, PCA-VAE; **b**, PCA-AE; **c**, PCA-VD; **d**, PCA-D; **e**, AE-PCA. AE, autoencoder; D, decoder network; VAE, variational autoencoder; VD, variational inference with a decoder network.

The five proposed PCA–DL configurations were developed according to these two design strategies (Fig. 3). This figure presents block diagrams that clearly illustrate the structural differences and data-flow pathways across each configuration. The hybrid configurations encompass two distinct architectural variants. The Wrapper architecture (PCA-VAE and PCA-AE) employs a fixed PCA projection as a rudimentary pre- and post-processing step that brackets the DL network. Conversely, the Bottleneck architecture (AE-PCA) inserts an adaptive, per-batch PCA operation within the AE's latent space, dynamically optimizing the representation. The integrated configurations (PCA-VD and PCA-D) are defined by their streamlined architecture, which eliminates the nonlinear encoder entirely and feeds the bottom networks directly from a frozen PCA projection.

The specific technical parameters for each of the five proposed configurations are detailed (Table 1). The core PCA function (PCA_SVD_) is implemented using SVD as defined by equations (2), (3), (4). For most configurations, this function is set to reduce dimensionality while preserving 95% of the accumulated variance. However, the AE-PCA configuration treats the dimensionality of its internal PCA bottleneck as a tunable hyperparameter, analogous to the number of hidden units in a neural network. A detailed theoretical defense of this model's structural stability and robustness against the choice of this hyperparameter is provided in the Supplementary Text Section S1. While all deep networks are built from standard fully connected (dense) layers with nonlinear activation functions, several networks incorporate specialized layers to enable their unique architectures. Both variational configurations (PCA-VAE and PCA-VD) use a lambda layer to implement reparameterization for sampling from the latent distribution. Furthermore, because PCA-VD lacks an encoder, it requires an auxiliary input layer to feed the raw data directly into its custom loss function (equation (6)). Finally, the AE-PCA model employs a batch normalization layer to standardize the latent variables before they are processed by its internal PCA.

**Table 1:** Structural details of the proposed configurations and benchmarks.

Category	Name	Encoder layer types	Decoder layer types
Proposed configurations	PCA-VAE	PCA_SVD_ (0.95), Input, Dense, Activation, Dense, Dense, Lambda	Latent Input, Dense, Activation, Dense, Activation, Loss, PCA_SVD_ (Inverse)
	PCA-AE	PCA_SVD_ (0.95), Input, Dense, Activation, Dense, Activation	Dense, Activation, Dense, Activation, PCA_SVD_ (Inverse)
	PCA-VD	PCA_SVD_ (0.95), Input, Dense, Dense, Lambda	Latent Input, Dense, Activation, Dense, Activation, Raw data, Loss
	PCA-D	PCA_SVD_ (0.95), Input	Dense, Activation, Dense, Activation
	AE-PCA	Input, Dense, Activation, Batch Normalization, PCA_SVD_	PCA_SVD_ (Inverse), Dense, Activation, Dense, Activation
Benchmarks	PCA	CPU-based PCA_SVD_ (0.95)	CPU-based PCA_SVD_ (Inverse)
	PCA	GPU-based PCA_SVD_ (0.95)	GPU-based PCA_SVD_ (Inverse)
	AE	Input, Dense, Activation, Dense, Activation	Dense, Activation, Dense, Activation
	VAE	Input, Dense, Activation, Dense, Dense, Lambda	Latent Input, Dense, Activation, Dense, Activation, Loss

Abbreviations: AE, autoencoder; CPU, central processing unit; D, decoder network; GPU, graphics processing units; PCA, principal component analysis; SVD, singular value decomposition; VAE, variational autoencoder; VD, variational inference with a decoder network.

**Fig. 4:**
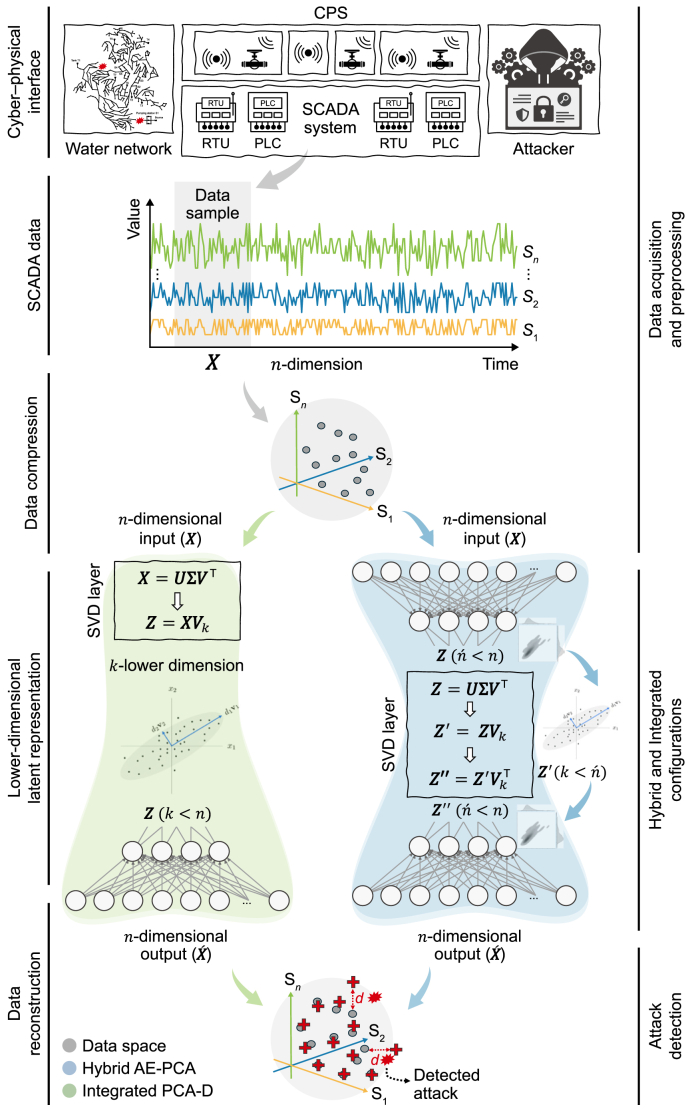
Schematic of the integrated PCA-D and hybrid AE-PCA for cyber–physical attack detection applied to cyber–physical systems (CPS) within water distribution systems (WDS). AE, autoencoder; D, decoder network; PCA, principal component analysis; PLC, programmable logic controller; RTU, remote terminal unit; SCADA, supervisory control and data acquisition; SVD, singular value decomposition.

A detailed schematic example depicts the operational data flow, using AE-PCA and PCA-D as representative models for the hybridization and integration strategies, respectively (Fig. 4). The AE-PCA model follows a multi-stage reconstruction process. Its nonlinear encoder first maps SCADA data into a high-dimensional latent representation. A batch normalization operation standardizes this representation, which is then subjected to a secondary, linear dimensionality reduction operation via the adaptive PCA bottleneck. This adaptive, per-batch PCA dynamically filters low-energy variance within the lower-dimensional representation. The resulting compressed representation is then orthogonally projected back into latent coordinates using the top-k singular vectors ($V_{k}V_{k}^{⊤}$) before being decoded to reconstruct the original data space. In contrast, the PCA-D model directly feeds PCA scores into the decoder via the fixed projection and reconstructs the original inputs without any inverse transformation step. This simplified architecture reduces the parameter count and nominal computational complexity. However, it forces the decoder to reconstruct the signal from a purely linear, rather than a learned nonlinear, representation.

## 3 Evaluation framework

This section details the comprehensive framework for systematically evaluating the five proposed PCA–DL configurations. The analysis is grounded by a direct comparison against three well-understood, standalone benchmarks: PCA, AE, and VAE. The performance of all configurations is assessed across two distinct WDS datasets representing simulated and real-world attack scenarios (Section 3.1). This evaluation proceeds through three stages: quantifying reconstruction performance (Section 3.2), assessing anomaly detection effectiveness (Section 3.3), and analyzing the associated computational costs (Section 3.4). Finally, these disparate metrics are translated into sustainability costs and synthesized into a single, holistic CER (Section 3.5), providing a definitive framework for model selection.

### 3.1 Datasets

An empirical evaluation of the proposed configurations is conducted across two distinct and challenging datasets, each representing a unique operational scenario. This dual-scenario approach is designed to rigorously test model robustness and generalizability. The scenarios include the BATADAL (BATtle of the Attack Detection ALgorithms) dataset [12] (Scenario I) and WADI (Water Distribution testbed) dataset [66] (Scenario II).

#### 3.1.1 Scenario I

The BATADAL dataset, developed for an international attack detection competition [12], provides a comprehensive set of WDS data explicitly designed for benchmarking security algorithms. The dataset contains 31 sensors and 12 actuators, including both normal operational data and a series of simulated attacks such as sensor manipulation or unauthorized changes to pump and valve states. Its high data collection frequency and extensive coverage of operational parameters make it an ideal environment for evaluating model performance under controlled, high-stress conditions.

#### 3.1.2 Scenario II

The WADI dataset is derived from a realistic, scaled-down WDS testbed designed to execute a range of cyber–physical attacks under real-world conditions [66]. This dataset encompasses a wide array of operational data, including high-resolution logs of sensor and actuator readings that capture the complex dynamics of water flow, pressure, and quality. It is recorded at a 1-s resolution, including both normal operations and a series of staged attack scenarios. The granularity of these data provides a rich source for validating the robustness of anomaly detection systems against more subtle, staged attack scenarios.

#### 3.1.3 Data preprocessing

Prior to model training, a standardized preprocessing protocol was applied to both datasets. Non-informative features with zero variance were removed, and partial missing values were imputed using forward-fill interpolation to maintain data continuity while introducing negligible artificial variance. All features were then standardized by removing the mean and scaling to unit variance, a process applied individually to the training and testing sets to prevent information leakage. After preprocessing, the BATADAL dataset (Scenario I) consisted of 39 features with 8761 training and 2089 testing samples. The WADI dataset (Scenario II) included 98 features with 1,209,601 training and 172,801 testing samples. Crucially, the testing sets differed in their attack density, with attack samples constituting approximately 19.5% of Scenario I, compared with only 5.7% of Scenario II.

### 3.2 Reconstruction performance criteria

To quantify the reconstruction performance of each model, three standard metrics were used: root-mean-squared error (RMSE, $e_{\text{rms}}$), mean absolute error (MAE, $e_{\text{abs}}$), and the coefficient of determination (*R*^2^) [67]. RMSE and MAE both measure the magnitude of the reconstruction error, while RMSE is more sensitive to large deviations due to its squaring term, making it particularly relevant for identifying the sharp error spikes caused by anomalies. *R*^2^ provides a normalized measure of the goodness of fit, indicating how well a model's reconstructions account for the variance in the original data. The mathematical formulations of these metrics are as follows:

(7) $$e_{\text{rms}}=\sqrt{\frac{1}{n}\sum_{i=1}^{n}{\left(x_{i}-\hat{x_{i}}\right)}^{2}}$$

(8) $$e_{\text{abs}}=\frac{1}{n}\sum_{i=1}^{n}\left|x_{i}-\hat{x_{i}}\right|$$

(9) $$R^{2}=1-\frac{\sum_{i=1}^{n}{\left(x_{i}-\hat{x_{i}}\right)}^{2}}{\sum_{i=1}^{n}{\left(x_{i}-\bar{x}\right)}^{2}}$$

where $x_{i}$ is the original data point, $\hat{x_{i}}$ is the reconstructed data point, $\bar{x}$ indicates the mean value of the original data, and n denotes the number of data points.

### 3.3 Detection performance criteria

The detection effectiveness of each configuration is evaluated using two primary analytical methods: confusion matrix analysis and receiver operating characteristic (ROC) curve analysis [68]. The confusion matrix classifies the identified anomalies against the ground-truth labels into four outcomes: true positives (TP), false positives (FP), false negatives (FN), and true negatives (TN). While accuracy (A) is calculated for completeness, the primary evaluation focuses on precision (P), recall (R), F1 score ($F_{1}$), and the area under the ROC curve (AUC), as these measures provide a more robust and operationally meaningful assessment of a model's ability to identify rare attack events correctly. These metrics are defined as follows [68].

Accuracy measures overall correctness relative to the total number of samples (N) in the confusion matrix:

(10) $$A=\frac{N_{\text{TP}}+N_{\text{TN}}}{N_{\text{TP}}+N_{\text{TN}}+N_{\text{FP}}+N_{\text{FN}}}$$

However, for highly imbalanced WDS datasets, accuracy can yield deceptively high values and is therefore an unreliable indicator of detection capability. Consequently, precision and recall are prioritized to assess operational reliability. Precision quantifies the reliability of an alert, where high precision values minimize false alarms and mitigate alarm fatigue among utility operators:

(11) $$P=\frac{N_{\text{TP}}}{N_{\text{TP}}+N_{\text{FP}}}$$

Recall measures the ability to detect all actual attacks (true positive rate, TPR). In CIP, high recall is vital to minimize missed threats that could lead to catastrophic physical consequences:

(12) $$R=\frac{N_{\text{TP}}}{N_{\text{TP}}+N_{\text{FN}}}$$

To provide a balanced measure of effectiveness across uneven class distributions, the F1 score is calculated as the harmonic mean of precision and recall:

(13) $$F_{1}=\frac{{2\times N}_{\text{TP}}}{{2\times N}_{\text{TP}}+N_{\text{FP}}+N_{\text{FN}}}$$

(14) $$F_{1}=2\times \frac{P\times R}{P+R}$$

The rate of false alarms is further quantified by the false positive rate (FPR, α), where a low FPR is essential to minimize the economic and labor costs of investigating nonexistent threats:

(15) $$\alpha =\frac{N_{\text{FP}}}{N_{\text{FP}}+N_{\text{TN}}}$$

Finally, AUC provides a threshold-independent assessment of the model's ability to discriminate between normal and anomalous instances across a range of operational sensitivities. This robust measure facilitates the comparison of detector reliability before a specific operating point is established.

Nonetheless, a consistent operating point is essential for a standardized comparison across all configurations. For all models, the anomaly detection threshold ($T_{\text{SPE}}$) was set at the 99th percentile of the SPE from the training dataset. This non-parametric threshold was selected for its robustness and to establish a conservative benchmark for comparing diverse architectures without model-specific tuning. Crucially, this statistical threshold is distinct from a formal economic cost function that weighs the operational penalties of FPs versus FNs. The analysis of those misclassification costs is captured by the precision and recall metrics, and the implications of this specific threshold choice are rigorously tested in the sensitivity analysis presented in Section 4.5.

### 3.4 Computational environment and metrics

All models were developed and evaluated on a Linux-based system equipped with an NVIDIA GeForce RTX 4090 GPU (24 GB video random-access memory), Intel Core i9-10920X central processing unit (CPU), and 64 GB of random-access memory. The DL architectures were constructed using Keras 3.4.1 with a TensorFlow backend, leveraging NVIDIA Compute Unified Device Architecture (CUDA, v12.6) for GPU acceleration. The $f_{\text{SVD}}$ functions were implemented to run on both GPU and CPU using the CuPy and NumPy libraries, respectively, to allow for a direct comparison of hardware acceleration. The final models’ structures and hyperparameters, determined using a random search protocol with early stopping, are detailed for each scenario in the Supplementary Tables S1 and S2.

To ensure the reproducibility of all computational metrics, the operational envelopes for both the hardware and its environment were standardized. All experiments were conducted in a temperature-controlled office environment, with the ambient temperature maintained at 24 °C (±1 °C). All CPU power limits (PL1/PL2) were set to their stock values in the system firmware, with standard demand-based frequency scaling enabled, to ensure consistent boosting behavior. Similarly, the GPU was configured via its system settings to its stock 100% power limit and a fixed thermal target of 84 °C. Under these standardized power and thermal constraints, the hardware's native boosting algorithms result in a repeatable average operating frequency characteristic of each benchmarked workload. Therefore, the different average frequencies observed for each model do not represent random fluctuations, but a direct reflection of the distinct computational intensity of each architectural configuration.

The computational cost of each configuration is quantified by monitoring resource utilization and execution time across three main stages: (i) model training, (ii) anomaly threshold computation, and (iii) the operational phase in which testing data are reconstructed. Two categories of metrics are recorded. Resource utilization metrics include GPU and CPU processor frequencies (GHz) and memory allocation (GB). Temporal metrics include user time, system time, and wall-clock time (minutes). A detailed description of these metrics and the monitoring tools is provided in the Supplementary Text Section S2.

### 3.5 Cost-effectiveness analysis

To move beyond a simple comparison of performance metrics and computational costs, this paper proposes a multi-stage framework for cost-effectiveness analysis. This framework provides an insightful, real-world assessment of DL-driven CPS for the security and sustainability of critical infrastructure. It first translates the measured computational loads into tangible sustainability costs and then normalizes these costs based on the models’ proven detection effectiveness to derive a single, unified comparative score.

#### 3.5.1 Energy analysis

The computational energy consumption for each configuration can be modeled by estimating the instantaneous power draw of the primary processing components and the baseline power draw of the workstation system [69]. The system's power consumption ($P_{\text{sys}}$) consists of both static (baseline) and dynamic (load-dependent) elements. Based on pilot measurements indicating minimal variability in baseline power and the negligible contribution of system memory (approximately 0.3–0.4 W GB^−1^), these components were excluded from the energy analysis for clarity and focus. Consequently, the instantaneous $P_{\text{sys}}$ for a given computational workload is approximated by scaling the nominal thermal design power (TDP, $P_{\text{TD}}$) of the CPU and GPU by their observed operating frequencies [70] as follows:

(16) $$P_{\text{sys}}\approx P_{\text{TD},\text{CPU}}\times \frac{v_{\text{inst},\text{CPU}}}{v_{\max,\text{CPU}}}+P_{\text{TD},\text{GPU}}\times \frac{v_{\text{inst},\text{GPU}}}{v_{\max,\text{GPU}}}$$

where $v_{\text{inst}}$ and $v_{\max}$ are the observed and maximum rated frequencies (GHz), respectively. In this study, the nominal TDP values were taken as $P_{\text{TD},\text{CPU}}$ = 165 W (at $v_{\max,\text{CPU}}$ = 3.5 GHz) and $P_{\text{TD},\text{GPU}}$ = 400 W (at $v_{\max,\text{GPU}}$ = 2.52 GHz). This power model is then used to calculate the total energy consumed during each experimental stage. The stage-specific energy consumption ($E_{s}$; in kJ) is derived from the measured wall-clock time ($T_{s}$; in seconds) and average observed processor frequencies ($\bar{v}$) as

(17) $$E_{s}=\frac{T_{s}}{1000}\sum_{i\in \left\{\text{GPU},\text{CPU}\right\}}P_{\text{TD},i}\times \frac{{\bar{v}}_{i,s}}{v_{\max,i}}$$

where ${\bar{v}}_{i,s}$ is the average observed frequency (GHz) of processor i during stage s. The annual energy requirement for continuous real-time monitoring ($E_{\text{RT}}$) is then extrapolated from the testing-stage energy consumption as follows:

(18) $$E_{\text{RT}}=E_{\text{test}}\times \frac{N_{s}}{N_{\text{sample}}}$$

where $N_{s}$ is equal to $3.1557\times 10^{7}$ (s yr^−1^) and $N_{\text{sample}}$ denotes the number of temporal samples during the testing stage. Finally, assuming two model development cycles per year, the total annual energy requirement ($E_{\text{total}}$) is determined as

(19) $$E_{\text{total}}=2\times \left(E_{\text{train}}+E_{\text{thr}}\right)+E_{\text{RT}}$$

The resulting $E_{\text{total}}$ value serves as the primary input for overall environmental impact analysis, while the $E_{\text{RT}}$ value is used to compare operational cost-effectiveness.

#### 3.5.2 Environmental impact analysis

The estimated annual energy requirement ($E_{\text{total}}$) is used to quantify two primary environmental impacts: greenhouse gas (GHG) emissions and water footprint (WF). To extrapolate these findings to a realistic data center deployment, the power usage effectiveness (PUE, $\pi_{\text{PUE}}$), which accounts for energy overheads such as cooling and lighting, must be considered. For this analysis, $\pi_{\text{PUE}}$ = 1.43 is assumed, consistent with the reported global PUE value for 2023 [33]. The corresponding GHG emissions ($m_{\text{CO}2}$) are estimated in kilograms of carbon dioxide equivalent (kg CO_2_eq) based on the electrical demand as follows:

(20) $$m_{\text{CO}2}=\pi_{\text{PUE}}\times E_{\text{total}}\times \frac{1}{3600}\times \frac{\gamma_{\text{grid}}}{\eta_{\text{PSU}}}$$

where $\eta_{PSU}$ is the efficiency of the power supply unit (assumed to be between 80% and 96%) [71] and $\gamma_{\text{grid}}$ is the grid-mix carbon emission factor (kg CO_2_eq kWh^−1^). This calculation quantifies the overall carbon intensity of each configuration under different deployment regions.

To complement this analysis, the WF of each configuration ($V_{w}$) is also estimated. This metric distinguishes between direct, on-site water usage for cooling and indirect, off-site water embedded in the generation of grid electricity [39] as follows:

(21) $$V_{w}=E_{\text{total}}\times \frac{1}{3600}\times \left[\omega_{\text{direct}}+\frac{\pi_{\text{PUE}}\times \omega_{\text{indirect}}}{\eta_{\text{PSU}}}\right]$$

where $\omega_{\text{direct}}$ is the site-reported water usage effectiveness (liters of cooling/humidification water per delivered computational energy in kWh^−1^) and $\omega_{\text{indirect}}$ is the water intensity of grid electricity (L kWh^−1^). The environmental factors ($\gamma_{\text{grid}}$, $\omega_{\text{direct}}$, and $\omega_{\text{indirect}}$) were evaluated across lower-, median-, and upper-bound ranges (Table 2).

**Table 2:** Summary of empirical factors for environmental impact analysis.

Factor	Lower	Median	Upper	Description	Ref.
$\pi_{\text{PUE}}$	1.1	1.43	1.8	Metric of data center overhead; lower bound reflects high-efficiency facilities (ideal = 1.0).	[33]
$\gamma_{\text{grid}}$ (kg CO_2_eq kWh^−1^)	0.02	0.465	1.1678	This factor varies significantly by region, with lower values corresponding to grids with high renewable energy penetration	[72]
$\omega_{\text{direct}}$ (L kWh^−1^)	0	0.5	2.0	Average ≈ 1.8; lower bound reflects air- or dry-cooled	[39]
$\omega_{\text{indirect}}$ (L kWh^−1^)	0.05	2.0	3.8	Average ≈ 2.18 for United States grid; lower bound wind/solar; upper bound reflects water-cooled thermoelectric generation	[40]

#### 3.5.3 Cost-effectiveness ratio (CER)

To synthesize the competing factors of sustainability cost and detection performance into a single, unified metric, this study formulates and applies a novel CER. This metric quantifies the real-world resource cost required to achieve one correctly identified anomaly (a TP). It is calculated by dividing the projected annual cost by the total number of annual TP detections:

(22) $$\kappa_{c}=\frac{C_{\text{ann}}}{N_{\text{sample}}\times \rho \times R},\text{where}\;C_{\text{ann}}\in \left\{E_{\text{elc}},m_{\text{CO}2},V_{w}\right\}$$

Here, $\kappa_{c}$ denotes CER for cost type c, and the numerator $C_{\text{ann}}$ represents the corresponding annual cost, taken as operational electricity requirement ($E_{\text{elc}}=\pi_{\text{PUE}}{\times E}_{\text{RT}}/\eta_{\text{PSU}})$, overall GHG emissions, or total WF. The denominator is the estimated number of annual TPs, where $N_{\text{sample}}$ is the number of processed samples per year, ρ is the expected anomaly rate, and R is the model's empirically measured recall, or detection effectiveness. Therefore, a lower CER value indicates superior cost-effectiveness, signifying a lower resource cost for each successfully detected anomaly.

However, it is critical to contextualize this metric properly. The CER is intentionally formulated to quantify the resource cost of a successful detection (a TP), providing a direct link between sustainability factors and security outcomes. This measure is not designed as a standalone decision metric but as a novel axis of evaluation to be considered alongside traditional performance criteria. The operational costs associated with misclassifications—namely, the security risks of FNs and the operational burden of FPs—are captured by the recall and precision metrics, respectively (Section 3.3). Consequently, the CER must be interpreted alongside the F1 score and *R*^2^ to form a holistic, multi-objective assessment in Section 4.4. By isolating the operational cost per TP, the CER provides a clear, interpretable measure of efficiency that complements, rather than replaces, the analysis of the full confusion matrix, thereby providing a more comprehensive framework for selecting genuinely sustainable security solutions.

## 4 Results and discussion

The evaluation results demonstrate that combining statistical methods with DL successfully disrupts the traditional tradeoff between detection effectiveness and computational cost. The central findings of this analysis are synthesized in the cost–performance frontier (Section 4.4), which visualizes the multi-objective performance of each PCA–DL configuration in terms of its detection effectiveness, reconstruction fidelity, and operational CER. The proposed framework shows that specific integrated and hybrid configurations achieved a notably more favorable balance compared with standalone methods, pushing beyond the established performance frontier either by substantially reducing resource consumption while preserving performance or by achieving superior detection accuracy at justifiable operational costs. The following subsections systematically deconstruct these findings by examining reconstruction performance (Section 4.1), detection effectiveness (Section 4.2), and the computational costs (Section 4.3), culminating in the cost-effectiveness analysis (Section 4.4) and its methodological validation (Section 4.5).

### 4.1 Impact on reconstruction performance

The integrated PCA-D and hybrid AE-PCA configurations consistently demonstrated superior reconstruction accuracy and robustness across both evaluation scenarios. Specifically, in the more complex Scenario II (WADI dataset), AE-PCA achieved the highest reconstruction fidelity (*R*^2^ = 0.887), lowest RMSE (0.308), and lowest MAE (0.110), closely followed by PCA-D (*R*^2^ = 0.880, RMSE = 0.317, MAE = 0.119). Despite notable differences in dataset complexity and attack frequencies, both models maintained superior performance relative to conventional standalone baselines (PCA, AE, VAE), validating their reliability and scalability.

Detailed quantitative metrics, including RMSE, MAE, and *R*^2^, as well as anomaly detection thresholds ($T_{\text{SPE}}$), are provided (Table 3). Across all evaluated configurations, strong generalization from training to testing was evident in consistently high *R*^2^ values and relatively modest increases in reconstruction error metrics. A persistent difference between RMSE and MAE further indicates that reconstruction errors primarily originated from significant deviations during attack periods, rather than fundamental fitting deficiencies. While the strong correlations among all reconstruction metrics confirm that the observed differences stem from architectural design rather than tuning bias, the results also underscore how combining PCA's robust generalization with DL's expressive power yields superior reconstruction.

**Table 3:** Reconstruction performance metrics and anomaly detection thresholds for the developed models using combined and standalone configurations under Scenarios I and II.

Configs	Scenario I							Scenario II						
	Train				Test			Train				Test		
	RMSE	MAE	R^2^	$T_{\text{SPE}}$	RMSE	MAE	R^2^	RMSE	MAE	R^2^	$T_{\text{SPE}}$	RMSE	MAE	R^2^
PCA-VAE	0.484	0.218	0.688	13.49	0.542	0.261	0.656	0.382	0.209	0.803	67.24	0.486	0.242	0.747
PCA-AE	0.271	0.157	0.850	8.827	0.437	0.215	0.761	0.286	0.158	0.867	47.79	0.401	0.184	0.822
PCA-VD	0.383	0.180	0.776	7.873	0.455	0.223	0.742	0.348	0.186	0.828	59.56	0.462	0.225	0.770
PCA-D	0.104^a^	0.061^a^	0.912^a^	2.347^a^	0.358^a^	0.133^a^	0.821^a^	0.146^b^	0.073^b^	0.928^b^	14.49^a^	0.317^b^	0.119^b^	0.880^b^
AE-PCA	0.168^c^	0.097^c^	0.895^c^	4.911^c^	0.420^c^	0.178^c^	0.774^c^	0.143^a^	0.070^a^	0.929^a^	15.67^b^	0.308^a^	0.110^a^	0.887^a^
d PCA	0.210	0.124	0.879	6.002	0.388^b^	0.176^b^	0.798^b^	0.210	0.118	0.905	28.43	0.351^c^	0.152	0.859^c^
d AE	0.163^b^	0.097^b^	0.896^b^	4.817^b^	0.446	0.197	0.750	0.187^c^	0.102^c^	0.914^c^	24.85^c^	0.366	0.147^c^	0.847
d VAE	0.344	0.165	0.805	6.731	0.476	0.231	0.723	0.360	0.200	0.820	59.65	0.489	0.245	0.743

Abbreviations: AE, autoencoder; Configs, configurations; D, decoder network; MAE, mean absolute error; PCA, principal component analysis; R^2^, coefficient of determination; RMSE, root-mean-squared error; SPE, squared prediction error; VAE, variational autoencoder; VD, variational inference with a decoder network.

$T_{\text{SPE}}$ is the SPE threshold calculated using q=0.99 on the training data.

a Best value in column.

b Second-best value in column.

c Third-best value in column.

d Conventional standalone methods used as benchmark models.

The analysis of $T_{\mathrm{SPE}}$, which is calculated at the 99th percentile of SPE derived from the training data, reinforces these insights. Specifically, lower $T_{\mathrm{SPE}}$ values observed for PCA-D (14.49) and AE-PCA (15.67) in Scenario II, compared with substantially higher thresholds for AE (24.85) and VAE (59.65), reflect a more accurate and tighter fit to normal operational data. Additionally, the inherent complexity of the datasets clearly influenced $T_{\mathrm{SPE}}$. Scenario II consistently exhibited higher thresholds (ranging from 14.49 to 67.24) compared with Scenario I (ranging from 2.35 to 13.49), which is primarily attributable to Scenario II's higher dimensionality (98 versus 39 features). Conversely, Scenario I exhibited greater degradation in reconstruction performance during testing, largely due to its substantially higher proportion of attack samples (19.5% compared with 5.7% in Scenario II). For example, PCA-D's RMSE increased by a factor of 3.4 in Scenario I but only by a factor of 2.2 in Scenario II, highlighting the pronounced impact of denser attack environments on reconstruction performance.

**Fig. 5:**
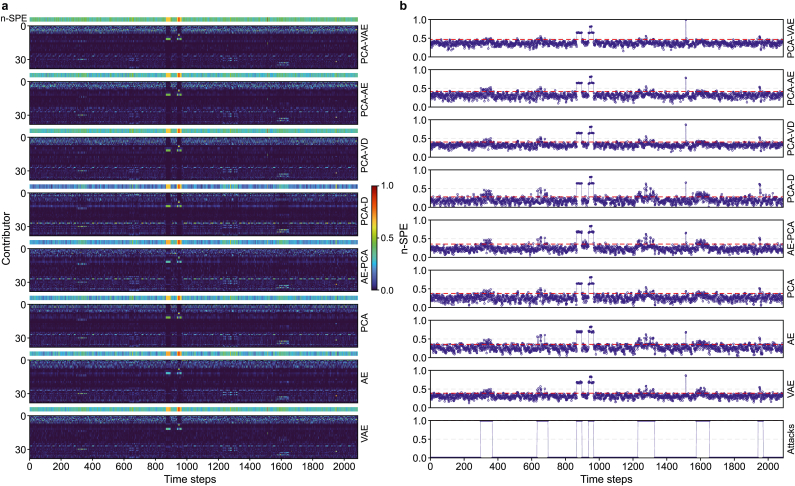
Reconstruction performance for all configurations in Scenario I. **a**, Heatmaps of feature-wise contributions to the normalized squared prediction error (n-SPE); **b**, Time series of n-SPE (solid lines with circular markers) overlaid with the corresponding detection thresholds (dotted lines).

**Fig. 6:**
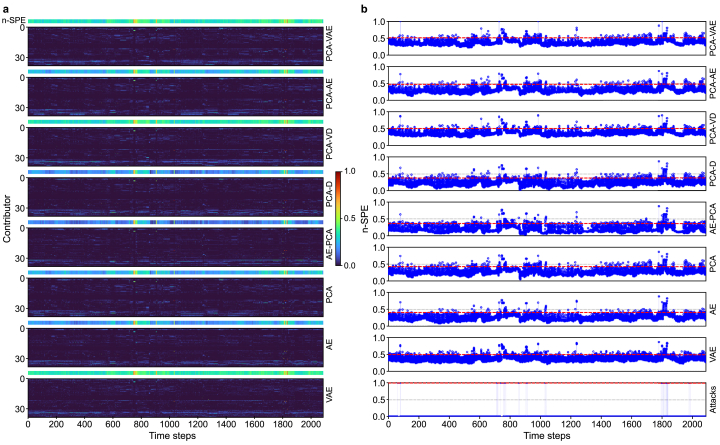
Reconstruction performance for all configurations in Scenario II. **a**, Heatmaps of feature-wise contributions to the normalized squared prediction error (n-SPE); **b**, Time series of n-SPE (solid lines with circular markers) overlaid with the corresponding detection thresholds (dotted lines).

A qualitative assessment of reconstruction errors (Fig. 5, Fig. 6) provides a compelling visual confirmation of the quantitative results. For each figure, the heat map illustrates the relative contribution of individual features to the reconstruction error, while the corresponding time series plot shows the magnitude of normalized SPE (n-SPE) versus the documented attack periods. In the attack-intensive Scenario I (Fig. 5), the superiority of the PCA-D and AE-PCA configurations is immediately apparent. They isolate anomalies into distinct error bands and generate sharp n-SPE spikes that noticeably exceed the detection threshold, while normal operational data remain correctly suppressed. This effectiveness is even more pronounced in the high-dimensional setting of Scenario II (Fig. 6), where both models render attack signatures as unambiguous spikes relative to a clean, low-error baseline. However, this visual analysis also reveals a critical tradeoff. The exceptionally clean baseline of the PCA-D model comes at the cost of missing several mid-sequence attacks, a limitation not observed in the more robust AE-PCA configuration.

This visual performance gap illuminates the representational tradeoff inherent to the integration strategy. While the standalone AE achieves robust reconstruction (Scenario II, RMSE = 0.366), switching to the PCA-D integration incurs a specific loss of modeling capacity. The AE's nonlinear encoder learns a task-relevant manifold, enabling it to capture low-variance but structurally significant nonlinear couplings—such as subtle hydraulic constraints—that characterize complex WDS operations. In contrast, the PCA-D architecture enforces a fixed unsupervised representation, discarding these low-energy interactions based solely on variance ranking. Consequently, what is “lost” in the PCA-D integration is the capacity to represent these subtle, nonlinear manifold dependencies. This loss of representational power explains why AE-PCA (which incorporates the nonlinear encoder) captures the mid-sequence attack signatures, whereas the linearized PCA-D suppresses them in the reconstruction-error space (Fig. 6).

A further examination of the baseline models clarified the strategic value behind hybrid and integrated approaches. The standalone AE model exhibited strong reconstruction fidelity during training (Scenario II, *R*^2^ = 0.914) but markedly lower performance on unseen test data (*R*^2^ = 0.847), indicating a tendency toward overfitting. Conversely, PCA models exhibited robust generalization capabilities (Scenario II, test-set *R*^2^ = 0.859) but with less expressive reconstruction power. This contrast highlights a fundamental tradeoff, where an AE yields detailed but overfitted reconstructions versus PCA's generalized but less detailed representations. Successful integration in the PCA-D and AE-PCA configurations addressed this dilemma by combining PCA's generalization strengths with AE's expressive capabilities, delivering consistently superior reconstruction accuracy and robustness.

In summary, the empirical outcomes strongly affirm the substantial advantages of integrated PCA-D and hybrid AE-PCA models. These results underscore the critical importance of strategic architectural design and lay a firm foundation for subsequent evaluations of anomaly detection effectiveness and computational efficiency.

### 4.2 Impact on anomaly detection effectiveness

The evaluation of anomaly detection effectiveness revealed a complex hierarchy of performance, a reality obscured by simplistic metrics such as accuracy. Due to the significant class imbalance inherent in the datasets, all models achieved deceptively high accuracy values. Therefore, this metric is insufficiently sensitive for this analysis. Consequently, the following discussion focuses on the interplay between precision, recall, F1 score, and AUC. Across these critical metrics, a trio of configurations, namely the hybrid AE-PCA and integrated PCA-D and PCA-VD, consistently outperformed all other models, each demonstrating distinct advantages depending on the operational scenario and performance objective.

**Fig. 7:**
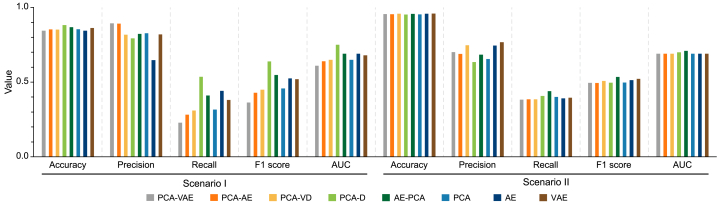
Comparison of the detection performance of the models based on the various configurations in terms of accuracy, precision, recall, F1 score, and AUC in Scenarios I and II, where a higher value indicates better performance. AE, autoencoder; AUC, area under curve; D, decoder network; PCA, principal component analysis; VAE, variational autoencoder; VD, variational inference with a decoder network.

Comprehensive quantitative results supporting these findings are summarized (Table 4) and visually compared (Fig. 7). Under the stress testing conditions in Scenario I, which are characterized by high attack frequency, the integrated PCA-D achieved the highest recall (0.536), F1 score (0.639), and AUC (0.750) among all evaluated configurations. Although hybrid configurations such as PCA-VAE and PCA-AE achieved high precision (0.894 and 0.891, respectively), their critically reduced recall limited their overall effectiveness due to frequent missed anomalies. This reduced recall resulted from their elevated $T_{\mathrm{SPE}}$, which failed to identify the majority of attacks. The operational tradeoff is starkly quantified by the raw detection counts. While PCA-VAE and PCA-AE identified only 93 and 115 TPs, respectively, AE-PCA and PCA-D identified 167 and 218, respectively. This gain in detected attacks came at a highly efficient price, yielding an average of 2.6 additional TP per extra FP and demonstrating a highly manageable and operationally robust detection strategy.

**Table 4:** Detection performance of the models based on the various configurations in terms of accuracy, precision, recall, F1 score, and AUC in Scenarios I and II.

Configurations	Scenario I					Scenario II				
	Accuracy	Precision	Recall	F1 score	AUC	Accuracy	Precision	Recall	F1 score	AUC
PCA-VAE	0.844	0.894	0.229	0.364	0.610	0.955	0.701	0.383	0.495	0.690
PCA-AE	0.854	0.891	0.283	0.429	0.640	0.955	0.689	0.385	0.494	0.690
PCA-VD	0.852	0.818	0.310	0.449	0.650	0.957	0.747	0.385	0.508	0.690
PCA-D	0.882	0.793	0.536	0.639	0.750	0.953	0.635	0.407	0.496	0.700
AE-PCA	0.868	0.823	0.410	0.548	0.690	0.956	0.683	0.440	0.535	0.710
a PCA	0.854	0.827	0.317	0.458	0.650	0.954	0.655	0.401	0.497	0.690
a AE	0.844	0.647	0.442	0.526	0.690	0.958	0.745	0.392	0.513	0.690
a VAE	0.863	0.820	0.381	0.520	0.680	0.959	0.768	0.396	0.523	0.690

Abbreviations: AE, autoencoder; AUC, area under curve; D, decoder network; PCA, principal component analysis; VAE, variational autoencoder; VD, variational inference with a decoder network.

a Conventional standalone methods used as benchmark models.

In the more realistic operational conditions in Scenario II, which are characterized by higher data complexity and fewer attack events, the performance rankings shifted slightly (Fig. 7). The hybrid AE-PCA configuration emerged as the most robust performer, achieving the highest recall (0.440), F1 score (0.535), and AUC (0.710). This outcome contrasts with PCA-D, whose effectiveness diminished in this scenario. The integrated PCA-VD offered a better balance of precision (0.747) and recall (0.385) than PCA-D (0.635 versus 0.407), proving more effective under these conditions. However, the consistent, high-level performance of AE-PCA across both scenarios underscores its superior robustness and scalability, making it the preferred configuration for operational environments, where detecting infrequent but critical anomalies is the primary objective.

**Fig. 8:**
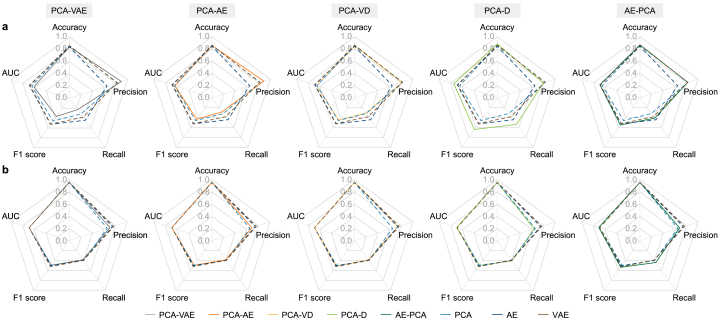
Comparison of the detection performance of the proposed and standalone configurations in terms of accuracy, precision, recall, F1 score, and AUC under Scenario I (**a**) and Scenario II (**b**). AE, autoencoder; AUC, area under curve; D, decoder network; PCA, principal component analysis; VAE, variational autoencoder; VD, variational inference with a decoder network.

A comprehensive synthesis of the design strategies (hybridization versus integration) provides important strategic insights (Fig. 8). The hybridization strategy yielded divergent outcomes. The unique AE-PCA configuration, which uses PCA as a structured bottleneck, proved highly effective by balancing FP and FN. Its performance polygon in the radar chart largely envelopes those of the baseline models, demonstrating a superior balance of F1 score and recall. Conversely, the cascaded hybrids (PCA-VAE and PCA-AE) consistently underperformed, with their polygons failing to enclose those of the standalone models, indicating a clear diminishment of detection capability. The integration strategies exhibited scenario-dependent strengths, with PCA-D excelling in the anomaly-dense environment of Scenario I and PCA-VD exhibiting more balanced and reliable performance under the high-dimensional, realistic conditions of Scenario II.

These strategic insights align well with established theories, confirming that anomaly detection effectiveness critically depends on the placement of the linear operator within the information pipeline. The “wrapper” hybrid strategy (PCA-AE or PCA-VAE) was found to be fundamentally inadequate because it applies a fixed, unsupervised linear projection prior to nonlinear processing, inadvertently discarding subtle yet task-relevant low-variance features. Conversely, the AE-PCA hybrid's effectiveness is attributable to its per-batch adaptive PCA bottleneck (Table 1). In this configuration, the encoder first maps input data onto a rich, task-aligned latent manifold. The subsequent batch-specific PCA projection, followed by its inverse transform, acts as a dynamic, low-rank denoiser. This per-batch projection provides the decoder with an adaptable, noise-suppressed latent space, reducing the residual complexity it must model and sharpening the contrast between normal and anomalous signals. The integration strategy is effective when anomaly variance aligns closely with the principal subspace retained by PCA, accounting for the observed scenario-dependent performance variations. While the deterministic PCA-D excelled in attack-dense scenarios (Scenario I), the probabilistic PCA-VD leveraged its rich posterior sampling to reconstruct partial signals from discarded dimensions in Scenario II. Finally, the standalone benchmarks, including the complex VAE, largely underperformed relative to the combined strategies, in line with prior observations [85]. The inherent design of the VAE, which is primarily focused on capturing normal data variability, caused it to “fail silently” with catastrophic consequences for recall.

From this analysis, two robust design principles emerge: (i) an adaptive, latent-space projector (AE-PCA) for broad operational robustness and (ii) a simpler PCA-to-decoder integration (PCA-D/VD) for computationally efficient, scenario-specific anomaly detection. Conversely, wrapping PCA with static, linear pre- and post-processing stages without adaptive mechanisms is conclusively identified as the least effective approach.

### 4.3 Impact on computational cost

The analysis of computational loads reveals that the integration strategy incurs a fundamentally lower cost than the hybridization strategy across all metrics. However, this reduction in cost must be weighed against the superior anomaly detection robustness of the leading hybrid model (AE-PCA), as established in Section 4.2. This core tradeoff positions computational cost as a critical factor in deploying AI-driven monitoring systems for sustainable urban infrastructure, particularly across deployment contexts where resources are constrained for real-time operation or frequent model retraining is necessary.

The analysis of execution times identifies two primary factors driving high computational cost: the prolonged training durations of complex hybrid models and the computational overhead of iterative variational inference during operation. The former is most evident when the workflow shifts from the compact, simulated data of Scenario I to the higher-dimensional, real testbed data of Scenario II. The discrepancy in training costs is evident (Table 5), where the training wall time for the hybrid AE-PCA model escalates from 1.65 min in Scenario I to 175.95 min in Scenario II. This disproportionate surge is attributable to its per-mini-batch SVD layer. The SVD kernel is arithmetic-light but GPU-inefficient and must be re-launched at every optimization step, inflating training time with increasing data complexity. In direct contrast, the integrated PCA-D and standalone AE models, which lack this specific bottleneck, converged in only 18.73 and 18.17 min, respectively, in the same scenario. This ∼9.4-fold increase in execution time reveals a critical tradeoff: the superior detection effectiveness of an architecturally complex model such as AE-PCA comes at a prohibitive development cost, rendering it unsuitable for agile deployment in urban infrastructure systems that require frequent retraining.

**Table 5:** Resource utilization and execution times measured during model training, threshold setting, and operational reconstruction under Scenarios I and II.

Configurations		Scenario I								Scenario II							
		GPU		CPU	Memory		Execution time			GPU		CPU	Memory		Execution time		
		Memory	Frequency	Frequency	Peak	Increment	User	Sys	Wall	Memory	Frequency	Frequency	Peak	Increment	User	Sys	Wall
		(GB)	(GHz)	(GHz)	(GB)	(GB)	(min)	(min)	(min)	(GB)	(GHz)	(GHz)	(GB)	(GB)	(min)	(min)	(min)
Stage (i)	PCA-VAE	21.20	1.23	0.32	1.89	0.34	0.60	0.15	0.66	22.30	2.29	0.18	12.28	1.01	13.98	4.37	14.73
	PCA-AE	21.10	2.29	0.32	1.87	0.34	3.27	1.17	3.23	22.20	2.32	0.18	5.94	2.33	47.65	18.20	47.60
	PCA-VD	21.30	1.23	0.14	2.21	0.07	0.60	0.18	0.65	22.20^a^	2.29^a^	0.14^a^	8.94^a^	1.01^a^	15.12^a^	5.48^a^	15.20^a^
	PCA-D	21.80^a^	1.13^a^	0.18^a^	1.96^a^	0.41^a^	1.07^a^	0.33^a^	1.02^a^	22.30	2.24	0.18	6.16	2.11	19.53	8.02	18.73
	AE-PCA	21.00^a^	2.22^a^	0.25^a^	1.92^a^	0.37^a^	1.53^a^	0.49^a^	1.65^a^	21.30^a^	2.49^a^	0.14^a^	5.22^a^	1.17^a^	136.47^a^	52.45^a^	175.95^a^
	PCA on CPU	0.00	0.00	0.42	0.78	0.00	0.01	0.02	0.01	0.00	0.00	3.43	6.40	3.54	1.37	0.81	0.13
	PCA on GPU	0.60	1.51	0.14	0.96	0.96	0.01	0.00	0.02	6.70	2.52	0.14	4.01	1.16	0.02	0.01	0.03
	AE	21.30^a^	1.46^a^	0.21^a^	1.86^a^	0.32^a^	1.07^a^	0.30^a^	0.98^a^	21.40	2.34	0.18	5.00	0.93	18.43	6.87	18.17
	VAE	21.20	0.78	0.18	2.06	0.51	1.02	0.13	0.58	21.40^a^	2.29^a^	0.18^a^	4.76^a^	0.59^a^	3.25^a^	0.83^a^	2.97^a^
Stage (ii)	PCA-VAE	21.30	1.13	0.25	1.92	0.02	0.17	0.04	0.19	22.30	2.29	0.14	13.83	2.67	18.23	5.17	18.77
	PCA-AE	21.20	1.01	0.25	1.90	0.03	0.04	0.01	0.04	22.20	2.29	0.14	7.73	2.14	1.70	0.47	1.60
	PCA-VD	21.30	1.18	0.14	2.22	0.02	0.23	0.06	0.25	22.20^a^	1.23^a^	0.14^a^	20.78^a^	13.11^a^	26.65^a^	7.42^a^	28.18^a^
	PCA-D	21.60^a^	0.53^a^	0.11^a^	1.97^a^	0.01^a^	0.02^a^	0.01^a^	0.03^a^	22.30	2.22	0.18	7.39	1.29	1.93	0.55	1.97
	AE-PCA	21.00^a^	2.14^a^	0.21^a^	1.96^a^	0.05^a^	0.09^a^	0.02^a^	0.07^a^	21.30^a^	2.34^a^	0.21^a^	6.16^a^	0.95^a^	7.12^a^	2.42^a^	8.92^a^
	PCA on CPU	0.00	0.00	0.35	0.79	0.01	0.01	0.01	0.01	0.00	0.00	1.26	5.42	1.66	0.12	0.05	0.02
	PCA on GPU	0.60	0.13	0.11	0.96	0.00	0.00	0.00	0.01	6.70	0.25	0.11	4.01	0.00	0.01	0.00	0.01
	AE	21.30^a^	0.73^a^	0.11^a^	1.86^a^	0.01^a^	0.03^a^	0.01^a^	0.04^a^	21.40	2.29	0.14	6.16	1.17	1.83	0.50	1.83
	VAE	21.20	2.14	0.07	2.09	0.03	0.22	0.05	0.20	21.40^a^	2.32^a^	0.14^a^	18.07^a^	13.33^a^	19.18^a^	5.83^a^	19.95^a^
Stage (iii)	PCA-VAE	21.20	0.91	0.25	1.93	0.02	0.07	0.02	0.08	22.30	2.29	0.14	12.26	1.08	2.92	0.82	3.03
	PCA-AE	21.20	0.15	0.14	1.90	0.00	0.01	0.00	0.02	22.20	2.29	0.14	7.92	0.18	0.33	0.09	0.34
	PCA-VD	21.30	0.98	0.11	2.22	0.01	0.08	0.02	0.08	22.20^a^	1.23^a^	0.14^a^	17.77^a^	0.99^a^	3.83^a^	1.05^a^	4.03^a^
	PCA-D	21.60^a^	0.15^a^	0.07^a^	1.91^a^	0.03^a^	0.02^a^	0.00^a^	0.02^a^	22.30	2.22	0.14	7.57	0.31	0.28	0.09	0.29
	AE-PCA	21.00^a^	1.74^a^	0.18^a^	1.99^a^	0.02^a^	0.07^a^	0.01^a^	0.03^a^	21.30^a^	2.34^a^	0.21^a^	6.22^a^	0.07^a^	0.94^a^	0.30^a^	1.15^a^
	PCA on CPU	0.00	0.00	0.07	0.79	0.00	0.00	0.00	0.01	0.00	0.00	0.67	5.60	0.18	0.02	0.03	0.01
	PCA on GPU	0.60	0.03	0.04	0.96	0.00	0.00	0.00	0.01	6.80	0.15	0.07	4.26	0.00	0.00	0.00	0.01
	AE	21.30^a^	0.15^a^	0.04^a^	1.90^a^	0.02^a^	0.02^a^	0.01^a^	0.03^a^	21.50	2.29	0.14	6.22	0.05	0.30	0.08	0.32
	VAE	21.20	0.96	0.07	2.05	0.02	0.09	0.02	0.08	21.40^a^	2.27^a^	0.14^a^	15.81^a^	1.68^a^	3.18^a^	0.96^a^	3.38^a^

Abbreviations: AE, autoencoder; CPU, central processing unit; D, decoder network; GPU, graphics processing units; PCA, principal component analysis; Sys, system; VAE, variational autoencoder; VD, variational inference with a decoder network.

a Entries corresponding to superior configurations identified in prior analyses.

Beyond training execution times, recurring computational costs during the operational phase are critical for practical deployment. The analysis of inference runtimes (Table 5, stage (iii)) reveals that the primary driver of this cost is the iterative sampling required by variational components, as demonstrated by two clear comparisons in Scenario II, where the integrated PCA-VD required 4.03 min for testing set reconstruction, representing a 14-fold increase over the 0.29 min required by its non-variational counterpart, PCA-D. This exact pattern is mirrored in the standalone baselines, where the VAE (3.38 min) was more than 10.6 times slower than the standard AE (0.32 min). This result proves that the Monte-Carlo procedure inherent to the variational architecture imposes an operational time cost that is an order of magnitude higher than that of the deterministic configurations, posing a severe challenge to its viability in real-time, resource-constrained systems.

Further analysis of execution times provides a clear baseline based on the standalone PCA models, highlighting the advantages of implementing hardware acceleration. This advantage is most evident in the complex Scenario II (98 features, 1.2 million observations), where the GPU-accelerated PCA completed training over four times faster than its CPU-based counterpart (0.03 min versus 0.13 min) (Table 5). GPU memory usage clustered around 21 GB across all DL-based configurations because CUDA pre-allocates memory to minimize fragmentation. In contrast, the CPU-bound PCA's clock speed jumped from 0.42 GHz to 3.43 GHz between scenarios, whereas the DL-based configurations exhibited no such variation, remaining firmly GPU dominated. This trend demonstrates that for high-dimensional data, GPU parallelism is not merely an enhancement but a fundamental requirement for achieving a tractable computational cost, even for the simplest linear models.

Synthesizing these findings reveals a clear hierarchy of computational cost driven by specific design choices. The hybridization strategy, exemplified by AE-PCA, imposes the highest development cost due to its extended training phase. The integration strategy, specifically PCA-D, drastically reduces this training cost to a level on par with standalone baselines. However, the use of variational components (PCA-VD) introduces a severe, recurring penalty to operational inference time. These distinct cost profiles are evident across all experimental stages, as reflected in processor frequency versus execution time (Supplementary Fig. S1). This observation underscores a fundamental tradeoff between the prolonged, resource-intensive training of the top hybrid model and the more balanced, rapid performance of the integrated configurations. A detailed breakdown of the resource utilization metrics, including GPU/CPU frequencies and memory usage for all stages and scenarios, further characterizes this architectural tradeoff (Supplementary Text Section S3).

In summary, computational cost is a multifaceted outcome, with this analysis showing that it is intricately linked to design strategy (integration versus hybridization), architectural elements (e.g., variational components or SVD layers), and hardware infrastructure (GPU versus CPU). These variations in computational load directly affect real-world operational costs, particularly annual electricity requirements and the corresponding environmental footprint. To identify the most truly sustainable and effective solution, these operational costs must be weighed against the detection performance established in Section 4.2. The following subsection synthesizes these disparate factors into a comprehensive cost-effectiveness analysis.

### 4.4 Cost-effectiveness

**Fig. 9:**
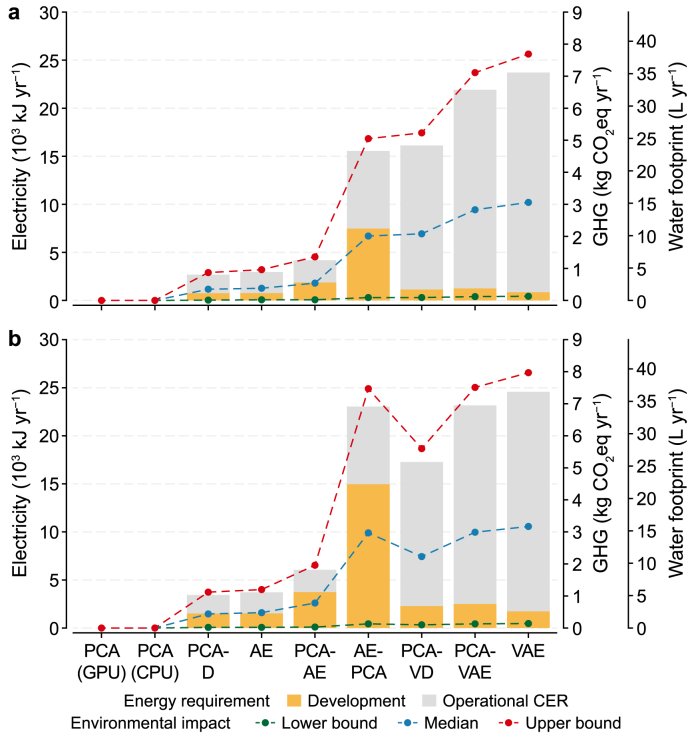
Comparison of annual energy requirements and associated environmental impacts for all configurations based on Scenario II under two operational strategies. **a**, A single annual model development cycle. **b**, Two annual model development cycles. Stacked bar charts show the total annual electricity requirement, separated into development and operational phases. Dashed lines plot the corresponding environmental impacts, including greenhouse gas (GHG) emissions and water footprint (WF). AE, autoencoder; CER, cost-effectiveness ratio; CPU, central processing unit; D, decoder network; GPU, graphics processing units; PCA, principal component analysis; VAE, variational autoencoder; VD, variational inference with a decoder network.

This final analysis translates the computational loads from Section 4.3 into annual energy and environmental costs, using Scenario II as a basis for extrapolation. This analysis reveals a vast disparity driven by architectural choices (Fig. 9). Under a single development cycle (Fig. 9a), the annual electricity requirement for the DL-based configuration spanned nearly an order of magnitude, ranging from an economical 2678 kJ for PCA-D, through 15,559 kJ for AE-PCA, to a high of 23,713 kJ for the standalone VAE, all of which substantially exceed the negligible sub-10-kJ cost of the baseline PCA models. The operational strategy of adding a second development cycle not only magnified these costs but also altered their relative ranking (Fig. 9b). One retraining cycle increased the energy requirement of the hybrid AE-PCA by 48.1%, while having only a marginal impact of 3.7% on the VAE. These electrical demands propagate directly to environmental impacts. Under the single-cycle baseline, the VAE's median carbon footprint (3.06 kg CO_2_eq) and water consumption (15.13 L) are approximately nine times higher than the 0.35 kg CO_2_eq and 1.71 L of PCA-D. This finding confirms that architectural decisions made during configuration design are a crucial factor in achieving system-level sustainability, as they directly dictate operational costs.

**Fig. 10:**
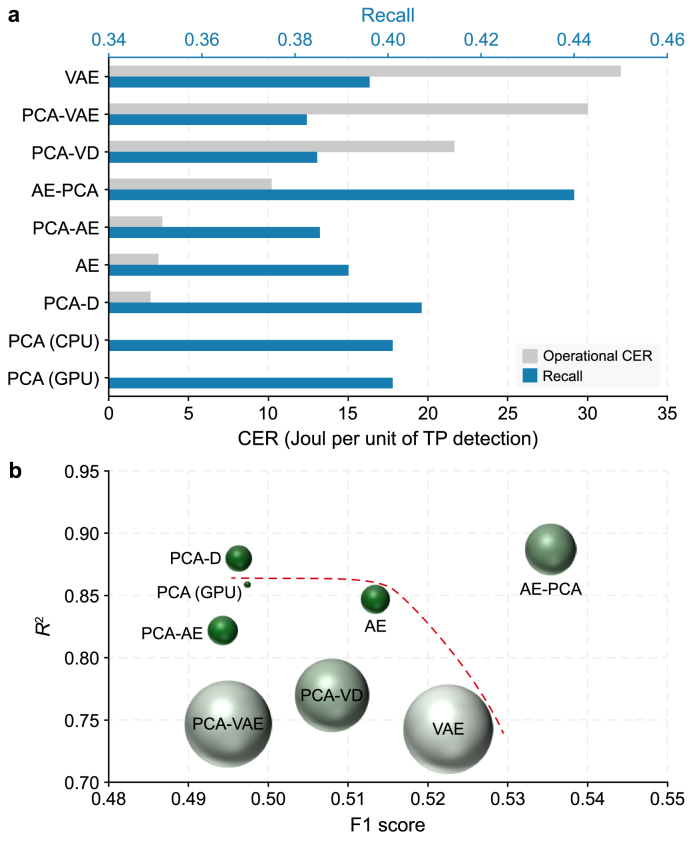
Cost-effectiveness and multi-objective performance analysis. **a**, Comparison of operational cost-effectiveness ratio (CER) and recall values. Grey bars represent the operational CER, where lower values are better; blue bars indicate the recall score, with higher values indicating better performance. **b**, Projection of model performance onto the F1-score-versus-*R*^2^ plane. The size of each marker is directly proportional to its operational CER, with larger markers indicating higher operational costs. The dashed red line indicates the performance frontier established by the baseline models (PCA, AE, and VAE). AE, autoencoder; CPU, central processing unit; D, decoder network; GPU, graphics processing units; PCA, principal component analysis; VAE, variational autoencoder; VD, variational inference with a decoder network.

However, simple energy and environmental analyses are insufficient, as they ignore detection performance. To provide a more holistic assessment, the operational CER was calculated, normalizing the energy cost by the number of TP detections. This analysis revealed a wide performance spectrum, with the operational CER ranging from 2.62 J TP^−1^ for the integrated PCA-D to 32.09 J TP^−1^ for the standalone VAE, representing an approximately 12.2-fold increase in the resource cost per successful detection. The critical tradeoff between raw performance and resource efficiency is evident (Fig. 10a). Notably, the configuration with the highest detection recall (AE-PCA at 0.440) did not yield the best cost-effectiveness (10.21 J TP^−1^). Conversely, the most cost-effective model (PCA-D) achieved its efficiency with a slightly lower recall of 0.407. Therefore, the CER provides a more nuanced basis for sustainable operations, balancing operational costs against the tangible benefits of successful anomaly detection.

To dissect these tradeoffs further, each model's performance was projected onto a plane comparing detection effectiveness (F1 score) and reconstruction fidelity (*R*^2^). This analysis reveals a performance frontier defined by the baseline models, which illustrates their inherent architectural compromises (Fig. 10b). Against this benchmark, the hybrid AE-PCA configuration achieved a decisive Pareto improvement, pushing well beyond the frontier to achieve the highest combined F1 score (0.535) and *R*^2^ (0.887). However, this superior dual performance is accompanied by a substantial operational CER, as indicated by its larger marker size, cementing its status as a high-cost, high-performance solution. Conversely, the integrated variational models (PCA-VD and PCA-VAE) proved inefficient, lagging behind the performance frontier while incurring the highest operational costs.

Beyond identifying a single best configuration, this multi-objective analysis reveals the task-specific suitability of different models. For example, the integrated PCA-D, while offering a lower F1 score than the standalone AE (0.496 versus 0.513), delivered superior reconstruction fidelity (*R*^2^ = 0.880 versus 0.847) at a 16% lower operational cost. This positions PCA-D not as a primary anomaly detector but as a highly efficient and more accurate solution for reconstruction-oriented tasks such as missing data imputation or signal denoising. This tradeoff underscores a critical principle for sustainable operation: the most cost-effective solution depends on the primary engineering objective, whether it is threat detection or data reconstruction.

This multi-objective analysis provides clear deployment guidance, positioning the integrated PCA-D and hybrid AE-PCA as solutions for distinct operational contexts. For dynamic CPS that undergo frequent system updates or require regular model retraining, the integrated PCA-D offers notable benefits. Its reduced parameter count, simplified architecture, and fixed linear projection make it the superior choice for resource-constrained environments, where the computational budgets for model retraining are limited. Conversely, for static, high-consequence infrastructure assets, where detection robustness is paramount, the hybrid AE-PCA is the most appropriate configuration. Its nonlinear encoder and adaptive PCA bottleneck preserve the subtle, low-variance structures—such as those arising from the complex hydraulic dynamic characteristic of WDS—that are necessary to detect nuanced, mid-sequence attacks. Such advantages justify higher computational costs in environments where the penalty for a missed detection is unacceptably high. Therefore, PCA-D defines the frontier for cost-efficiency in evolving systems and high-signal regimes, whereas AE-PCA defines the frontier for robustness in complex systems and low-signal regimes.

The practical implications of these operational CER values are revealed in the total annual operational footprint for continuous monitoring under Scenario II baseline conditions. Regarding the median values of external factors, the efficient PCA-D (recall = 0.407) would detect 736,996 TPs annually, consuming 0.53 kWh of operational energy and incurring a footprint of 0.25 kg CO_2_eq and 1.22 L of water. In contrast, the more robust AE-PCA (recall = 0.440) would detect 790,923 TPs, but at a cost of 2.24 kWh, 1.04 kg CO_2_eq, and 5.15 L. This tangible, 4.2-fold increase in the annual environmental burden is the operational price for achieving a 7.3% increase in successful attack detection.

### 4.5 Sensitivity analysis

To test the robustness of the study's findings, a three-part sensitivity analysis was conducted. This analysis quantifies the impact of the $T_{\text{SPE}}$ operating point, operational anomaly rate (AR), and external environmental factors on the central conclusions of this study.

#### 4.5.1 Sensitivity to the anomaly detection threshold

First, the sensitivity of key performance metrics to the choice of the $T_{\text{SPE}}$ operating point was evaluated. This analysis for the two leading configurations (AE-PCA and PCA-D) is summarized across five distinct SPE percentiles used to define $T_{\text{SPE}}$ (Table 6).

**Table 6:** Sensitivity of detection performance and operational CER to $T_{\text{SPE}}$ operating point.

Model	Operating point (SPE percentile)	Resulting precision	Resulting recall	F1 score	Operational CER (J TP^−1^)
AE-PCA	85.0%	0.621	0.448	0.520	10.04
	95.0%	0.662	0.442	0.530	10.17
	98.0%	0.678	0.441	0.534	10.20
	99.0%	0.683	0.440	0.535	10.21
	99.5%	0.687	0.440	0.536	10.21
PCA-D	85.0%	0.584	0.439	0.501	2.43
	95.0%	0.623	0.425	0.505	2.51
	98.0%	0.628	0.415	0.500	2.57
	99.0%	0.631	0.407	0.498	2.62
	99.5%	0.633	0.407	0.498	2.62

Abbreviations: AE, autoencoder; CER, cost-effectiveness ratio; D, decoder network; PCA, principal component analysis; SPE, squared prediction error.

The results quantitatively confirm the fundamental precision–recall tradeoff (Table 6). As the SPE percentile used to set $T_{\text{SPE}}$ increases, precision consistently improves at the expense of recall. Because recall is in the denominator of the CER calculation (equation (22)), this decrease in detection effectiveness directly inflates the cost per true positive. For example, for the PCA-D model, tightening the operating point from the 85.0th percentile to the 99.5th percentile decreased recall by 7.3%, which in turn inflated the operational CER by 7.8%. This analysis confirms that cost-effectiveness is not a static property of a model but is fundamentally coupled to operational strategy. Although the 99.0th percentile represents a balanced operating point for comparison, any strategic deviation toward higher precision incurs a quantifiable security penalty (more missed attacks) and corresponding sustainability penalty (an inflated operational CER).

#### 4.5.2 Sensitivity to the anomaly rate (AR)

To further examine the dependence of the cost-effectiveness rankings on the operational AR, the operational CER for the two leading configurations (Table 7), calculated across three distinct AR conditions: 1.0%, 5.7% (the baseline rate from Scenario II), and 15.0%.

**Table 7:** Sensitivity of operational CER to anomaly rate (AR) conditions.

Model	AR condition	Assumed AR	Operational CER (J TP^−1^)
AE-PCA	Low	1.0%	58.21
	Baseline (WADI)	5.7%	10.21
	High	15.0%	3.88
PCA-D	Low	1.0%	14.80
	Baseline (WADI)	5.7%	2.62
	High	15.0%	0.99

Abbreviations: AE, autoencoder; CER, cost-effectiveness ratio; D, decoder network; PCA, principal component analysis; TP, true positives; WADI, water distribution testbed.

The relative cost-effectiveness ranking of the architectures remains stable across all conditions (Table 7). As expected, the absolute CER values are inversely proportional to the AR. However, the integrated PCA-D model consistently outperforms the hybrid AE-PCA model, maintaining a 3.9-fold cost-effectiveness advantage, regardless of the attack frequency. This finding is critical because it confirms that the superior cost-effectiveness of the PCA-D configuration is a fundamental property, rather than an artifact of the 5.7% AR in the WADI dataset (Scenario II).

#### 4.5.3 Sensitivity to external environmental factors

Finally, the analysis tested the robustness of the environmental cost rankings to variations in external deployment factors. The operational CER for energy, carbon, and water was calculated using the lower-bound, median, and upper-bound factors for $\pi_{\text{PUE}}$, $\gamma_{\text{grid}}$, $\omega_{\text{direct}}$, and $\omega_{\text{indirect}}$ (Table 2). The results for the two leading configurations are reported (Table 8).

**Table 8:** Sensitivity of operational CER to external environmental factors.

Model	Factor level	Operational CER^a^		
		(J TP^−1^)	(mg CO_2_eq TP^−1^)	(mL TP^−1^)
AE-PCA	Lower bound	7.86	0.04	0.11
	Median	10.21	1.32	6.52
	Upper bound	12.85	4.17	16.94
PCA-D	Lower bound	2.00	0.01	0.03
	Median	2.62	0.34	1.67
	Upper bound	3.27	1.06	4.31

Abbreviations: AE, autoencoder; CER, cost-effectiveness ratio; D, decoder network; PCA, principal component analysis; TP, true positives.

a CERs for carbon and water are presented in milligrams (mg) and milliliters (mL) for readability.

The results reveal two key findings. First, the absolute environmental costs are highly sensitive to external factors (Table 8). For example, the operational CER (in mg CO_2_eq TP^−1^) increases by more than two orders of magnitude between the lower-bound and upper-bound conditions. Second, despite this volatility, the relative ranking of the configurations remains stable (Table 8). The integrated PCA-D model is consistently more cost-effective than the hybrid AE-PCA model across all metrics and under all conditions. For every tested bound, PCA-D maintains an approximately four-fold advantage in energy, carbon, and water efficiency. This trend provides evidence that architectural efficiency is the dominant factor in environmental performance. Therefore, this conclusion is not an artifact of the median assumptions but a fundamental property of the models themselves.

It is imperative to contextualize these findings within the scope of a massive-scale, real-world deployment. The energy and environmental costs presented here are extrapolations from a single, controlled testbed. In a city-scale CPS comprising hundreds of thousands of sensor-instrumented nodes, the aggregate operational cost would be orders of magnitude higher, exposing a critical tradeoff at the heart of the sustainable smart city, where the operational intelligence gained from granular, real-time data comes at the cost of a substantial and continuous environmental burden. As highlighted by prior work, an AI-enabled WDS can generate up to 270 GB of data daily from a 2.7-million-meter water network [73], posing immense scalability and energy challenges. Therefore, while this analysis demonstrates the relative superiority of specific architectures, it also serves as a stark reminder that the absolute cost of smart CIP at scale remains a primary and formidable barrier to sustainable implementation.

## 5 Conclusions

This study examined the central paradox inherent to AI-driven CIP, namely the tension between security effectiveness and environmental sustainability. By proposing a comprehensive cost-effectiveness framework—centered on the novel CER metric—to systematically evaluate hybrid and integrated PCA–DL configurations, the presented analysis demonstrates that architectural design fundamentally shapes the tradeoff between performance and sustainability-related resource consumption. The empirical findings of this study lead to the following actionable recommendations for key stakeholders involved in the development of future sustainable and secure smart cities.

Comparative analyses highlighted that a one-size-fits-all approach to AI-driven CIP is both inefficient and operationally simplistic. Optimal model selection fundamentally depends on the operational objectives and risk profiles of specific CPS operations. For static, high-consequence infrastructure assets, where detection effectiveness is paramount, the hybrid AE-PCA configuration is the most appropriate. Despite its higher training costs, this one-time investment is justified by more balanced operational costs and superior recall and F1 score. Conversely, for dynamic CPS that undergo frequent system updates or require regular model retraining, the integrated PCA-D offers notable benefits. Its reduced parameter count and simplified architecture facilitate rapid and economically viable model redeployment. The multi-stage framework and CER presented in this paper equip utility managers with the quantitative tools needed to adopt a mission-specific, adaptive deployment strategy.

The growing reliance on AI-driven CPS for CIP in smart cities introduces significant but often overlooked environmental implications. Policymakers and urban planners must account for the long-term energy and resource demands of AI systems within strategic planning, budgeting, and procurement processes for smart infrastructure. This study offers a rigorous methodological framework for evaluating sustainability tradeoffs among competing DL-based methods in CIP contexts. Cities should encourage vendors to provide transparent, validated end-to-end resource consumption profiles, explicitly including training, inference, and retraining phases, and to report model cost-effectiveness using comprehensive metrics such as the proposed CER ($\kappa_{c}$). Incorporating these key criteria into public tenders can shift decision-making from mere purchasing to strategic governance aligned with broader sustainability goals.

The pronounced differences in environmental impacts among the evaluated models, driven primarily by architectural design, demonstrate that resource efficiency is fundamentally an engineering challenge that can be addressed systematically. This study directly challenges the prevailing yet overly simplistic approach in which PCA serves merely as a rudimentary preprocessing step. Empirical evidence from the wrapper architectures (PCA-AE and PCA-VAE) confirms that this perspective is not only suboptimal but also theoretically flawed for unseen anomaly detection, as it risks discarding precisely those low-variance signals that often indicate the most catastrophic threats. In contrast, the effectiveness of the bottleneck architecture (AE-PCA) and integrated PCA-D/VD models is underpinned by a critical architectural principle: in both designs, the decoder retains direct access to the original high-dimensional data space, allowing it to learn nonlinearities effectively, while PCA acts as an efficient linear subordinate. Consequently, the research community must elevate architectural frugality to a primary design principle in DL-driven cyber–physical security, adopting more holistic evaluation benchmarks such as the CER. Benchmarking challenges such as BATADAL should also broaden their evaluation criteria to explicitly reward the development and adoption of green AI.

Although this study provides a foundational framework for green AI in cyber–physical security, its specific scope defines several directions for future research. The analysis presented here focused on reconstruction-based methods for detecting unseen anomalies and employed foundational AE, VAE, and PCA to enable an interpretable comparison across different configurations and design strategies. Future research should apply this cost-effectiveness analysis to additional CIP methods, more advanced DL architectures, and model-centric optimization problems. A rigorous investigation of the dimensionality of the internal PCA bottleneck, particularly under data drift and varying anomaly sparsity, remains an important direction for future work aimed at real-world deployment. Additional investigation into other statistical methods beyond PCA is also warranted.

Furthermore, the empirical findings presented here rely exclusively on two WDS datasets, both of which are limited to anomalies caused by deliberate cyber–physical attacks. Although WDS serves as a robust proxy for many CPS, the generalization of hybridization and integration principles to other domains, such as electrical grids or gas networks, requires further investigation. Anomaly signatures in these systems may differ significantly. For example, the subtle, low-variance signals of a stealthy attack in a WDS may not map directly to the high-frequency transient signals of a grid fault. Therefore, future research should evaluate the generalizability of these architectural principles across a wider range of anomalies, including those arising from natural or accidental system failures, and explore other critical infrastructure domains, such as electrical grids, gas networks, and transportation networks. By continuing this line of research, subsequent studies can help establish more broadly generalizable green AI design principles and a new generation of genuinely sustainable AI solutions that secure critical infrastructure without compromising the environmental integrity of the smart cities they aim to protect.

## CRediT authorship contribution statement

**JungMin Lee:** Writing – original draft, Validation, Supervision, Resources, Methodology, Investigation, Funding acquisition, Formal analysis, Conceptualization. **Amir Saman Tayerani Charmchi:** Writing – review & editing, Writing – original draft, Visualization, Validation, Supervision, Software, Methodology, Investigation, Formal analysis, Data curation, Conceptualization. **Fatemeh Ghobadi:** Writing – original draft, Visualization, Validation, Project administration, Investigation, Formal analysis, Data curation, Conceptualization. **Myeong In Kim:** Writing – original draft, Validation, Project administration, Data curation.

## Declaration of competing interest

The authors declare that they have no known competing financial interests or personal relationships that could have appeared to influence the work reported in this paper.
