# Supplementary material for: Green AI architectures: Navigating the security–sustainability paradox in critical infrastructure protection
Source: Environ Sci Ecotechnol. 2026 Apr 6;31:100697. doi: 10.1016/j.ese.2026.100697 (PMC13091774; doi:10.1016/j.ese.2026.100697)
Supplement: Multimedia component 1 [file mmc1.docx]

**Green AI Architectures: Navigating the Security–Sustainability Paradox in Critical Infrastructure Protection**

*An online Supplementary file*

JungMin Lee^a,^*^,1^, Amir Saman Tayerani Charmchi^b,1^, Fatemeh Ghobadi^b,1^, Myeong In Kim^a^

^a^ Land and Housing Research Institute, Smart Climate Environment Research Center, Daejeon, South Korea

^b^ Digital Twin and Artificial Intelligence Research Lab, Digital Integration Department, Onpoom Corp. R&D Center, Seoul 07222, Republic of Korea

* Correspondence: Tel.+82-10-3360-4502; Email: andrew4502@lh.or.kr

^1^ These authors contributed equally to this work (Co-first authors).

**S.1. Model Architectures and Hyperparameters**

The dimensionality of the AE-PCA internal PCA bottleneck represents an influential hyperparameter whose structural stability warrants specific discussion prior to real-world deployment. Although a formal sensitivity analysis falls outside the present scope of this study, the model’s design provides inherent robustness.

In the AE-PCA architecture, the function of the internal PCA layer differs fundamentally from that of a global feature selector. The preceding nonlinear encoder first constructs a robust latent manifold that concentrates the normal data into a compact representation. Since the PCA layer operates after this feature extraction, and is recomputed per mini-batch, it functions primarily as a low-rank denoising operator. This division of labor ensures that the encoder retains the dominant structural burden. Consequently, modest variations in PCA dimensionality affect only how aggressively the batch-level noise is trimmed, rather than perturbing the underlying global manifold structure. This functional separation provides robustness against small parameter deviations, making the AE-PCA less sensitive to rank choice than architectures where PCA is applied directly to the raw, high-dimensional input (e.g., PCA-AE, PCA-VAE). Empirical observation during model tuning confirmed that performance stability is maintained around the chosen rank.

The following tables detail the final architectures and hyperparameters for all models tested in Scenario I (**Table S1**) and Scenario II (**Table S2**). These parameters were determined via a random search over a pre-defined hyperparameter space, beginning with simple architectures that were incrementally increased in complexity to maintain computational efficiency. To prevent overfitting, training was regularized using an early stopping criterion based on a shuffle-holdout validation set (20% of training data). The criterion monitored the validation loss (see main manuscript, **Eqs. 5** and **6**). It halted training after five epochs of no improvement, after which the weights from the best-performing epoch were restored.

**Table S1** Final model architectures and hyperparameters for Scenario I

| **Architectural Strategy** | **Model Name** | **Component** | **Layer (Output size, Activation)** | **Latent Space Dim** | **Parameters**  **(Trainable / Total)** | **Hyperparameters (Optimizer, Learning rate, Batch size)** |
| --- | --- | --- | --- | --- | --- | --- |
| **Hybridization (Wrapper)** | PCA-VAE | Encoder | Input (12) → Dense (12, relu) | z-mean: Dense (8) & z-log-var: Dense (8) | 628 / 628 | Adam, 0.001, 64 |
|  |  | Sampling | Lambda (8) |  |  |  |
|  |  | Decoder | Input (8) → Dense (12, relu) → Dense (12, linear) |  |  |  |
|  |  | Loss | Input layer, Decoder, z-mean & z-log-var → Functional (12) |  |  |  |
|  | PCA-AE | Encoder | Input (12) → Dense (12, relu) → Dense (8, relu) | 8 | 524 / 524 | Nadam, 0.001, 16 |
|  |  | Decoder | Input (8) → Dense (12, relu) → Dense (12, linear) |  |  |  |
| **Integration** | PCA-VD | Encoder | Input (12) | z-mean: Dense (16) & z-log-var: Dense (16) | 2,247 / 2,247 | Adam, 0.001, 64 |
|  |  | Sampling | Lambda (16) |  |  |  |
|  |  | Decoder | Input (16) → Dense (32, relu) → Dense (39, linear) |  |  |  |
|  |  | Loss | Original input, Decoder, z-mean & z-log-var → Functional (39) |  |  |  |
|  | PCA-D | Decoder | Input (12) → Dense (32, relu) → Dense (39, linear) | None | 1,703 / 1,703 | Nadam, 0.001, 64 |
| **Hybridization (Bottleneck)** | AE-PCA | Encoder | Input (39) → Dense (32, relu) | 8 | 3,687 / 3,751 | Adam, 0.001, 64 |
|  |  | Bottleneck | Batch Normalization → PCA (8) → PCA Inverse (32) |  |  |  |
|  |  | Decoder | Input (32) → Dense (32, relu) → Dense (39, linear) |  |  |  |
| **Standalone (Benchmark)** | AE | Encoder | Input (39) → Dense (32, relu) Dense (8, relu) | 8 | 3,119 / 3,119 | Nadam, 0.001, 64 |
|  |  | Decoder | Input (8) → Dense (32, relu) → Dense (39, linear) |  |  |  |
|  | VAE | Encoder | Input (39) → Dense (32, tanh) | z-mean: Dense (16) & z-log-var: Dense (16) | 4,167 / 4,167 | Nadam, 0.001, 256 |
|  |  | Sampling | Lambda (16) |  |  |  |
|  |  | Decoder | Input (16) → Dense (32, tanh) → Dense (39, linear) |  |  |  |
|  |  | Loss | Input layer, Decoder, z-mean & z-log-var → Functional (39) |  |  |  |

**Table S2** Final model architectures and hyperparameters for Scenario II

| **Architectural Strategy** | **Model Name** | **Component** | **Layer (Output size, Activation)** | **Latent Space Dim** | **Parameters**  **(Trainable / Total)** | **Hyperparameters (Optimizer, Learning rate, Batch size)** |
| --- | --- | --- | --- | --- | --- | --- |
| **Hybridization (Wrapper)** | PCA-VAE | Encoder | Input (45) → Dense (45, relu) | z-mean: Dense (32) & z-log-var: Dense (32) | 8,569 / 8,569 | Nadam, 0.001, 64 |
|  |  | Sampling | Lambda (32) |  |  |  |
|  |  | Decoder | Input (32) → Dense (45, relu) → Dense (45, linear) |  |  |  |
|  |  | Loss | Input layer, Decoder, z-mean & z-log-var → Functional (45) |  |  |  |
|  | PCA-AE | Encoder | Input (45) → Dense (45, relu) → Dense (32, relu) | 32 | 7,097 / 7,097 | Nadam, 0.001, 16 |
|  |  | Decoder | Input (32) → Dense (45, relu) → Dense (45, linear) |  |  |  |
| **Integration** | PCA-VD | Encoder | Input (45) | z-mean: Dense (32) & z-log-var: Dense (32) | 11,426 / 11,426 | Nadam, 0.001, 64 |
|  |  | Sampling | Lambda (32) |  |  |  |
|  |  | Decoder | Input (32) → Dense (64, relu) → Dense (98, linear) |  |  |  |
|  |  | Loss | Original input, Decoder, z-mean & z-log-var → Functional (98) |  |  |  |
|  | PCA-D | Decoder | Input (45) → Dense (64, relu) → Dense (98, linear) | None | 9,314 / 9,314 | Nadam, 0.001, 64 |
| **Hybridization (Bottleneck)** | AE-PCA | Encoder | Input (98) → Dense (64, relu) | 32 | 16,994 / 17,122 | Nadam, 0.001, 64 |
|  |  | Bottleneck | Batch Normalization → PCA (32) → PCA Inverse (64) |  |  |  |
|  |  | Decoder | Input (64) → Dense (64, relu) → Dense (98, linear) |  |  |  |
| **Standalone (Benchmark)** | AE | Encoder | Input (98) → Dense (64, relu) Dense (32, relu) | 32 | 16,898 / 16,898 | Nadam, 0.001, 64 |
|  |  | Decoder | Input (32) → Dense (64, relu) → Dense (98, linear) |  |  |  |
|  | VAE | Encoder | Input (98) → Dense (64, tanh) | z-mean: Dense (32) & z-log-var: Dense (32) | 18,978 / 18,978 | Nadam, 0.001, 256 |
|  |  | Sampling | Lambda (32) |  |  |  |
|  |  | Decoder | Input (32) → Dense (64, tanh) → Dense (98, linear) |  |  |  |
|  |  | Loss | Input layer, Decoder, z-mean & z-log-var → Functional (98) |  |  |  |

**S.2. Computational Cost Monitoring: Metrics and Tools**

This section details the metrics and software tools used to quantify the computational cost of each model configuration, as discussed in the main manuscript.

**S.2.1. Monitored Metrics**

The following two categories of metrics were recorded:

1. Resource utilization metrics:
   1. GPU/CPU processor frequency (GHz): The average clock speed of the primary processing unit during a given stage.
   2. Memory allocation (GB): The peak memory allocated on the relevant device (GPU VRAM or system RAM) during a stage.
2. Temporal metrics:
3. Wall-clock time (minutes): The total real-world time elapsed from the start to the end of a computational stage.
4. User time (minutes): The CPU time spent executing the user's code.
5. System time (minutes): The CPU time spent executing kernel-level operations on behalf of the user's code.

**S.2.2. Monitoring Tools**

All measurements were performed within the standardized hardware and environmental envelope detailed in **Section 3.4** of the main manuscript. The specific tools used for monitoring are as follows. GPU resource utilization (processor frequency, memory allocation) was monitored using the NVIDIA System Management Interface (*nvidia-smi*) command-line utility. CPU and system RAM utilization were monitored using the *psutil* library in Python and validated with the *%%memit* cell magic function from the *memory_profiler* IPython extension. All temporal metrics (wall-clock, user, and system time) were captured using the *%%time* cell magic function within the IPython/Jupyter environment.

**S.3 Detailed Analysis of Computational Costs**

This section provides a breakdown of the computational cost analysis, including the resource utilization and execution time metrics that support the summary findings presented in the main manuscript's **Section 4.3**. As illustrated in **Fig. S1**, which plots average GPU/CPU operating frequency against execution time, the configurations exhibit distinct cost profiles across all experimental stages. This visualization highlights the disparity between the resource-intensive training requirements of hybrid configurations and the streamlined operational performance of integrated architectures.


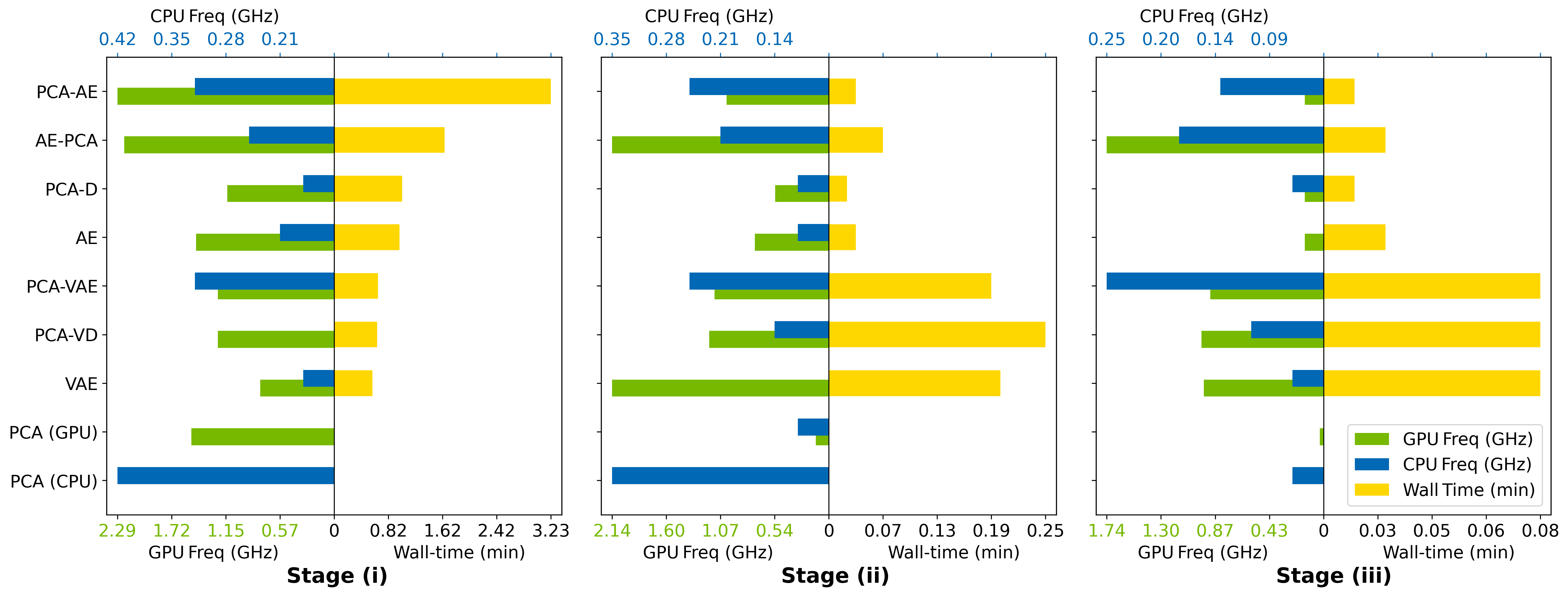


(a)


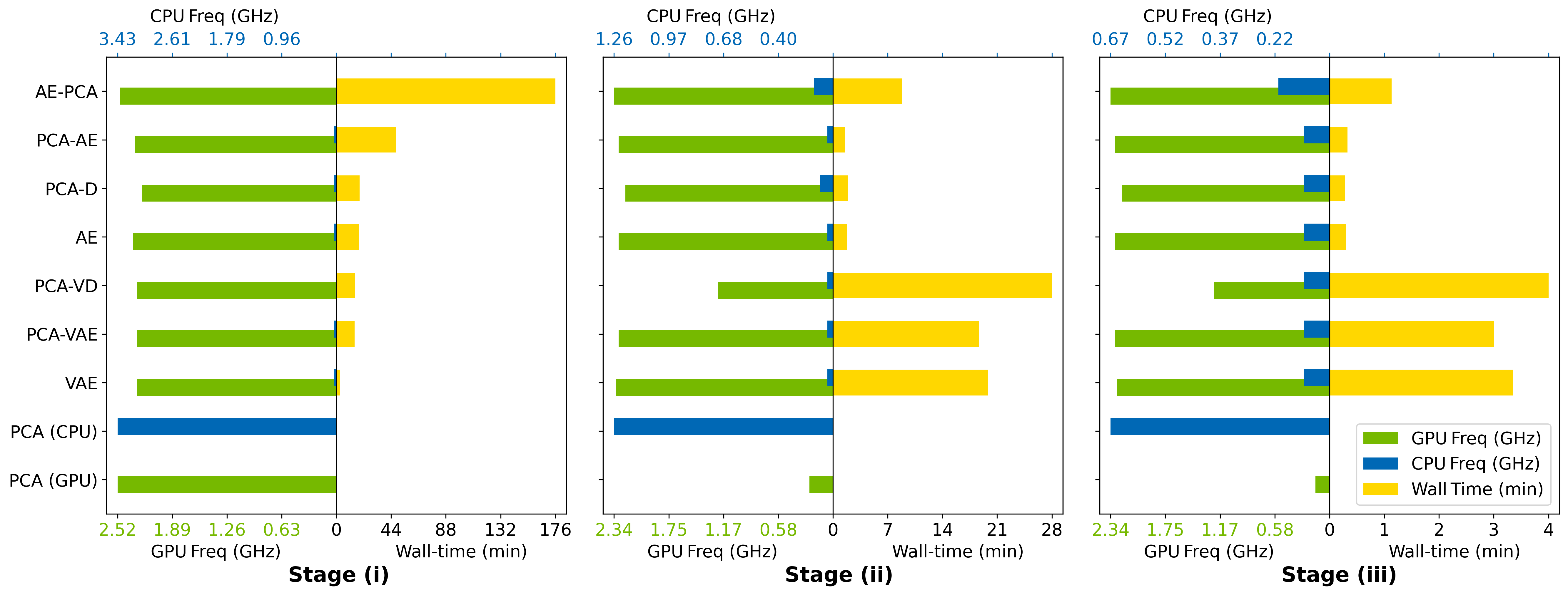


(b)

**Fig. S1.** Visual representation of CPU/GPU clock frequency against wall-clock execution time for each model and computational stage for: (a) Scenario I and (b) Scenario II.

Analysis of resource utilization reveals two key patterns. First, GPU memory usage was consistently high (~21.3 GB) for all deep learning (DL) configurations, a result of CUDA's memory pre-allocation strategy within the 24 GB physical memory limit. The exceptions were the GPU-accelerated PCA (using 0.6-6.8 GB) and the CPU-based PCA. Second, processor frequencies varied significantly with data complexity. The CPU-based PCA, being entirely CPU-bound, showed a substantial increase in CPU frequency between Scenarios I and II (e.g., from 0.42 GHz to 3.43 GHz during training). Conversely, the DL-based configurations remained GPU-bound with stable, low CPU frequencies, indicating their computational load is borne almost entirely by the GPU.

The frequent discrepancy between wall-clock and user times, particularly during training, indicates that most processes were not solely processor-bound and benefited from effective parallelization on multi-core processors. The transition from the simpler Scenario I to the high-dimensional Scenario II profoundly impacted computational loads. This was most dramatically illustrated by the AE-PCA model, whose training wall-time escalated by two orders of magnitude, from 1.65 to 175.95 minutes, highlighting how dataset complexity can create exponential, rather than linear, increases in computational cost for specific architectures.

A comparison of the model categories reveals the following performance trade-offs:

- Standalone models: The benchmarks established clear boundaries. GPU-accelerated PCA was by far the fastest baseline, completing training in Scenario II over four times faster than its CPU-based counterpart. In contrast, standalone AE and VAE models were orders of magnitude slower (up to 540 times longer than GPU-PCA), underscoring the immense architectural overhead of DL.
- Variational vs. deterministic models: A clear performance divide emerged based on model type. All configurations incorporating variational inference (VAE, PCA-VAE, PCA-VD) incurred a significant and recurring computational penalty during the operational and threshold-computation phases. This is attributed to the iterative Monte-Carlo sampling procedure required for reconstruction, making them inherently slower in production environments compared to their deterministic counterparts.
- Hybrid vs. integrated models: The proposed configurations also showed distinct profiles. Integrated models (PCA-D, PCA-VD) were generally more computationally balanced than their hybrid counterparts. The AE-PCA hybrid, while a top performer in detection effectiveness, consistently demanded the highest GPU and CPU resources, resulting in the longest execution times, particularly during its complex training stage.
